# Cardiac and Digestive Forms of Chagas Disease: An Update on Pathogenesis, Genetics, and Therapeutic Targets

**DOI:** 10.1155/mi/8862004

**Published:** 2025-04-21

**Authors:** Amanda Farage Frade, Hélléa Guérin, Joao Paulo Silva Nunes, Luiz Felipe Souza e Silva, Vinicius Moraes de Paiva Roda, Rafael Pedro Madeira, Pauline Brochet, Pauline Andrieux, Jorge Kalil, Christophe Chevillard, Edecio Cunha-Neto

**Affiliations:** ^1^Laboratory of Immunology, Heart Institute (InCor), University of São Paulo Medical School, São Paulo 05403-900, Brazil; ^2^French National Institute for Health and Medical Research (INSERM), UMR U1090, TAGC Theories and Approaches of Genomic Complexity, MarMaRa Institute, Aix Marseille University, Marseille 13288, France; ^3^Institute for Investigation in Immunology (III), National Institute of Science and Technology (INCT), São Paulo 05403-900, Brazil; ^4^Department of Clinical Immunology and Allergy, University of São Paulo Medical School, São Paulo 01246-903, Brazil

**Keywords:** cardiomyopathy, Chagas disease, digestive form, genetics, mitochondria, pathogenesis, therapy

## Abstract

Chagas disease, caused by the protozoan parasite *Trypanosoma cruzi* (*T. cruzi*), is a neglected disease affecting around 6 million people, with no effective antiparasitic drugs or vaccines. About 40% of Chagas disease patients develop symptomatic forms in the chronic phase of infection, chronic Chagas cardiomyopathy (CCC) or digestive forms like megaoesophagus and megacolon, while most infected patients (60%) remain asymptomatic (ASY) in the so-called indeterminate form (IF). CCC is an inflammatory cardiomyopathy that occurs decades after the initial infection. Death results from heart failure or arrhythmia in a subset of CCC patients. Myocardial fibrosis, inflammation, and mitochondrial dysfunction are involved in heart failure and arrhythmia. Survival in CCC is worse than in other cardiomyopathies. Distinct from other cardiomyopathies, CCC displays a helper T-cell type 1 (Th1-T) cell–rich myocarditis with abundant interferon-gamma (IFN-*γ*) and tumor necrosis factor-alpha (TNF-*α*) and selectively lower levels of mitochondrial energy metabolism enzymes and high-energy phosphates in the heart. A CD8+ T cell–rich inflammatory infiltrate has also been found in the Chagasic megaesophagus, which is associated with denervation of myoenteric plexi. IFN-*γ* and TNF-*α* signaling, which are constitutively upregulated in Chagas disease patients, negatively affect mitochondrial function and adenosine 5′-triphosphate (ATP) production–cytokine-induced mitochondrial dysfunction. In addition, the differential susceptibility to developing CCC has prompted many studies over the past 25 years on the association of genetic polymorphisms with disease outcomes. A comprehensive understanding of Chagas disease pathogenesis is crucial for identifying potential therapeutic targets. Genetic studies may offer valuable insights into factors with prognostic significance. In this review, we present an updated perspective on the pathogenesis and genetic factors associated with Chagas disease, emphasizing key studies that elucidate the differential progression of patients to CCC and other symptomatic forms. Furthermore, we explore the interplay between genetic susceptibility, inflammatory cytokines, mitochondrial dysfunction and discuss emerging therapeutic targets.

## 1. Introduction

Chagas disease is a tropical vector-borne neglected tropical disease caused by the protozoan parasite *Trypanosoma cruzi*, also known as American Trypanosomiasis. The parasite is transmitted by the triatomine insect vector in poor rural housing in endemic areas but can also be passed congenitally through blood transfusion, via organ transplant, or by oral ingestion [1].

Chagas disease affects an estimated 6–7 million people worldwide, the majority of whom reside in Latin America. Each year, ~30,000 new cases and 10,000 deaths are reported in this region, which remains the most heavily impacted by the disease. Additionally, 75 million people in the Americas are at risk of contracting the disease, with 9000 new cases annually resulting from mother-to-child transmission. Due to migration, the disease has also become a global health concern, with over 300,000 cases identified in the United States and Europe [2, 3].

Chagas disease is often asymptomatic (ASY) during the acute phase. However, in the chronic phase, which may occur 5–30 years after the initial infection, ~30% of patients develop symptomatic complications such as chronic Chagas cardiomyopathy (CCC) with electrocardiogram changes, including electric conduction defects, and 10% develop gastrointestinal disorders like megaesophagus or megacolon, or a combination of both [4]. The rest of the chronically infected patients (around 60%) remain ASY in the so-called indeterminate form (IF). Among the 30% that develop CCC, around 1/3 develop severe CCC, with life-threatening heart failure (left ventricular ejection fraction [EF] ≤ 0.4; mostly detected with heart ultrasound) and/or arrhythmia (mostly detected in electrocardiogram tracings and 24 h Holter recording), while two-thirds of CCC patients. CCC has a worse prognosis and is less responsive to heart failure or arrhythmia treatment than cardiomyopathies of noninflammatory etiology, like idiopathic or ischemic cardiomyopathies (ICs) [5]. Diagnosis of digestive disease in Chagasic patients with dysphagia or severe constipation is made with X-ray analysis of barium esophagography and manometry for megaesophagus and barium enema for megacolon.

Nifurtimox and benznidazole are the only drugs with proven efficacy against *T. cruzi* infection, but only in the acute phase of the disease or during childhood, but not in adults with chronic infection, a time when most patients are diagnosed, and there is no vaccine available [6]. Indeed, a randomized clinical trial with Benznidazole treatment failed to improve the progression of CCC in spite of reducing blood parasitism [7]. CCC is the most prevalent etiology of myocarditis in the world and the most important cause of non-IC in endemic regions of Latin America, where it stands as a major indication for heart transplantation. The annual mortality rate for the population with chronic Chagas heart disease is estimated at 4%, ranging from 1% to 10% according to risk stratification. In common with other cardiomyopathies, CCC shows myocardial hypertrophy and fibrosis. However, inflammation is particularly important in CCC. The clinical severity of Chagas disease is correlated with the occurrence of myocarditis [8].

The histopathological hallmark of CCC is persistent myocarditis and fibrosis, which can affect the electrical conduction system or the atrial and ventricular myocardium. This leads to pathological myocardial remodeling, conduction abnormalities, arrhythmias, or left ventricular dysfunction, often progressing to dilation and congestive heart failure. Death results from heart failure or arrhythmia, which develops in a subset of CCC patients. In the digestive form of the disease, inflammatory damage to the myenteric plexus with loss of neurons results in impaired peristalsis, causing stasis, dilation, and obstruction, manifesting as dysphagia (esophageal disease, megaesophagus) and/or severe constipation (dilation of the colon, megacolon).

Unfortunately, there is currently no vaccine or antiparasitic drug with proven efficacy for patients in the chronic phase, when most of them are diagnosed. It is estimated that only 10% of Chagas disease patients have been diagnosed.

## 2. Pathogenesis and Genetics of Symptomatic Forms of Chronic Chagas Disease

The intracellular life cycle of *T. cruzi* is a major target of the antiparasitic immune response [7]. Upon cell infection by *T. cruzi*, extracellular and endosomal Toll-like receptors (TLRs) are activated [9], triggering MyD88-mediated activation of nuclear factor kappa-light-chain-enhancer of activated B cells (NF-*κ*B) [10]. After escaping the endolysosomal compartment, *T. cruzi* differentiates into its replicative amastigote form in the cytoplasm, leading to inflammasome activation, which induces inflammatory cytokines and further NF-*κ*B activation [11]. This cascade promotes the release of proinflammatory cytokines, such as IL-12 and IL-18, in macrophages and dendritic cells, ultimately driving the differentiation of anti-*T. cruzi* interferon-gamma (IFN-*γ*)-producing Th1 cells [12]; IFN-*γ—*increases the destruction of *T. cruzi* in macrophages by increasing the synthesis of reactive oxygen species (ROS) and reactive nitrogen species (RNS). Most information about the immunopathology of the acute phase of Chagas disease comes from mouse models of *T. cruzi* infection, while knowledge about the immunopathology/pathophysiology of symptomatic forms of Chagas disease has been accrued in patients. Mice genetically deficient in pathogen resistance genes such as TLRs and TLR signaling components, IL-12 and IFN-*γ* have higher parasite loads and increased mortality upon acute *T. cruzi* infection, directly implicating such genes and pathways in protection against *T. cruzi* infection [13]. Additional immune effectors like anti-*T. cruzi* antibodies, CD8+ T cells, and natural killer cells (NK cells) strongly contribute to parasite control. Despite the robust immune response initiated during the acute phase of infection, a chronic infection often follows, characterized by low-grade persistent infection with very low parasitism in the presence of anti-*T. cruzi* antibodies, which can be assessed in serological tests [14]. Plasma IFN-*γ* and tumor necrosis factor-alpha (TNF-*α*) levels are increased in all clinical forms of chronic Chagas disease, even more so among CCC patients, who also have an increased number of circulating IFN-*γ*-producing CCR5+ and CXCR3+ Th1 cells [11, 12, 14], as compared to those with the IF of the disease [15, 16]. Given the scarcity of *T. cruzi* in the CCC heart tissue, heart-directed autoimmunity has been suggested as the driver of inflammation in myocarditis [17]. A body of data from our group and others in chronically *T. cruzi*-infected mice [18, 19], as well as in CCC patients, shows the presence of anti-cardiac myosin CD4+ T cells and autoantibodies that are cross-reactive to *T. cruzi* antigen B13. Immunization of mice with *T. cruzi* antigens induces a cardiac myosin-specific immune response [20], while sensitization of human peripheral blood cells with *T. cruzi* B13 protein was able to raise cardiac myosin-cross-reactive T-cell clones [21–23]. Heart-infiltrating T cells from CCC patients were found to massively produce IFN-*γ* [11, 24, 25]; this cytokine is among the most upregulated in Chagas hearts [26] and is the top upstream regulator of the gene expression profile in CCC heart tissue [27, 28]. Increased mRNA levels of Th1 T-cell markers CCR5 and CXCR3, as well as their chemokine ligands, are found in CCC heart tissue; mRNA levels of Th1 T cell–chemoattracting chemokine CXCL9 in CCC hearts were positively correlated with the intensity of myocarditis [29]. Taken together, this suggests that IFN-*γ* producing *T. cruzi* B13-specific CCR5+ and CXCR3+ Th1 cells sensitized in the periphery could migrate to the heart, where they would cross-reactively recognize cardiac myosin and/or other cardiac antigens, producing IFN-*γ* locally [26]. This could lead to excessive collateral damage by IFN-*γ*-producing T cells in the hearts of CCC patients, as described in acutely *T. cruzi*-infected mice. It has been reported that transgenic mice overexpressing IFN-*γ* in plasma develop a TNF-*α* dependent myocarditis and cardiomyopathy [30]. IFN-*γ* is thus considered the culprit of CCC [31].

Regarding the mechanism for the increased number of Th1 T cells in CCC patients, preliminary observations suggest that differentiation of naïve T cells into IFN-*γ*-producing Th1 T cells is favored on CCC as compared to IF patients. In addition, a body of data supports that Chagas disease patients who progress to CCC with ventricular dysfunction display an early impaired pathogen-killing immune response [32, 33], which could lead to the observed increase in parasitism among severe CCC patients (i.e., those with ventricular dysfunction) [32, 34, 35]. High parasitism, IFN-*γ*, and TNF-*α* plasma levels are all positively correlated with clinical severity of CCC [15, 36], while reduction of *T. cruzi* parasitism with benznidazole treatment leads to reduced plasma IFN-*γ* levels and IFN-*γ* producing T cells [37, 38]. Taken together, these findings may indicate that CCC progression might be associated with early impairment of a pathogen-killing immune response, leading to increased parasitism and, thus, parasite-induced IFN-*γ* production. This adds a layer of complexity to the role of excessive inflammation in CCC development.

CCC patients display reduced numbers of peripheral blood IL-10-producing regulatory T cells as compared to IF patients [12, 31, 39–41]. Moreover, CCC and mixed cardiac and digestive Chagas disease patients exhibited a lower number of CD14+/HLA-DRlow/− monocytes (myeloid-derived suppressor cells) [42] and a higher number of classical and intermediate monocytes associated with a more pronounced inflammatory environment, when compared to the ASY forms [43]. Chagasic megaesophagus patients display increased basal production of IFN-*γ* and increased TNF-*α*/IL-10 ratio on stimulated PBMC [44], and Chagas digestive disease patients showed increased mRNA expression of innate immunity genes TLR8 and IFN-*β*1 in PBMC, which was correlated with colonic and rectal size [45]. This suggests a deficiency in the regulatory arm of the immune response in CCC and the digestive forms of Chagas disease. Regarding local inflammation in digestive disease, inflammatory infiltrates rich in TIA-1 cytotoxic T cells, CD57+ NK cells, and CD68+ macrophages are found in myoenteric plexi, associated with extremely reduced neuron counts and detection of *T. cruzi* DNA among megaesophagus patients [46]. It has recently been shown that IFN-*γ* and T-bet expression, indicative of T1-type inflammation, is found in chagasic megaesophagus [47].

### 2.1. Genetic Susceptibility in Chagas Disease

Familial aggregation of CCC cases, case–control studies on candidate genes, and genome-wide association studies (GWASs) indicate a role of genetics in the development of symptomatic forms of Chagas disease [48]. More recently, the use of whole-exome sequencing in Chagas families or unrelated individuals disclosed rare or intermediate-frequency polymorphisms that could have a significant effect. This paper updates a previous review of genetic factors and pathogenesis of Chagas disease published in 2014 by our group, reporting 45 publications on gene polymorphisms associated with Chagas disease clinical forms from 1998 to 2014 [4].

In the candidate gene approach, the investigated genes have either been found to be involved in the immunity against *T. cruzi* infection in genetically deficient murine models of acute Chagas disease [13] and/or play a role in the pathophysiology of symptomatic chronic forms of the disease, studied in patients. Essentially all genes have genetic polymorphisms—that are DNA sequences in them that vary among different individuals and which may be associated with qualitative or quantitative changes in the encoded gene. In case–control studies, the frequencies of a given gene polymorphism (an allele or genotype) in a “case” cohort (for instance, CCC patients) are compared with frequencies in the “control” cohort (for instance, the ASY IF of Chagas disease) using chi-square or Fisher's exact test contingency tables, or stepwise binary logistic regression analysis, with sex and polymorphisms as covariates. It is important that all case and control subjects are genetically unrelated. Case–control studies have a retrospective cross-sectional design; that is, they compare two groups, one with an outcome and the other without it. When frequencies of a given allele or genotype are significantly higher (typically with a *p* value of less than 0.05) in the “case” than the “control” cohort, this implies that carrying the polymorphism increases the risk of developing the disease and thus the polymorphism is said to be associated with the disease. In this case, the odds of having the disease among carriers of the polymorphism—odds ratio (OR) and its 95% confidence interval (CI)—are above 1. ORs and 95% CI below 1 indicate that the polymorphism is associated with protection from developing disease. Some case–control studies have shown an association of a given gene allele/genotype with the acquisition of chronic infection (when comparing frequencies of gene polymorphisms between Chagas'disease patients—“cases” and seronegative, non-*T. cruzi*-infected “control” subjects) or with progression toward symptomatic Chagas disease (when comparing frequencies of gene polymorphisms between the CCC and/or digestive disease “cases” with ASY IF patients – “controls”). These gene variants can impact not only the disease progression from ASY to symptomatic forms but also its severity (when comparing frequencies of gene polymorphisms between severe CCC—that is, CCC with ventricular dysfunction—with mild/moderate CCC—that is, CCC with electrocardiogram changes but no ventricular dysfunction). The majority of the gene polymorphisms tested for association with Chagas disease have been previously described as associated with other inflammatory or infectious diseases, mostly in samples of Caucasian/European ancestry. Many of them have been described as functional polymorphisms—that is, the gene variant affects the function of the gene product (changing the amino acid sequence of the encoded protein) or quantitatively affects the expression levels of the gene (for instance, in variants occurring in nonprotein-coding regions of a gene with transcriptional regulatory activity). On the other hand, some studies were performed with the “tag SNP” approach, where tested SNPs are chosen to represent the whole genetic variability in that gene and are not necessarily functional polymorphisms [49–51].  Table 1 summarizes the case–control studies on gene polymorphisms conducted in Chagas disease patients since 1998 up to 2024, encompassing over 80 publications that examine variants in 73 distinct genes in cohorts coming from different Latin American endemic countries, published between 1998 and 2024. It can be observed in Table 1 that while associations of some gene variants are shared between cohorts of different countries, several of the associations that are observed in a given country are not replicated in a different country or in a different cohort in the same country. Gene polymorphism case/control association studies have several limitations and pitfalls that can lead to failure to replicate results and failure to inform causality. One of them is that the studied gene variants are “common” (that is, the minor or less frequent allele—minor allele frequency, or MAF—is present in around 10%–20% of the population). Each “common” polymorphism usually has a small (1%–10%) contribution to the phenotype, and this is reflected in modest strength of association, with most ORs between 1 and 3. For instance, an OR of 1.2 implies a 20% increase in the chance of getting the disease, while an OR of 3 implies a 3-fold increase in the chance of getting the disease. An additional complication is that an associated polymorphism is not necessarily the “causal” polymorphism. It may simply be a marker of a closely linked, undetected polymorphism that is truly causal—a situation called linkage disequilibrium. At any event, functional studies must be performed before the polymorphism is indeed causally related to the phenotype. Indeed, an additional challenge is the different ethnical composition and miscegenation of the cohorts in different Latin American countries. Most published genetic studies have been conducted in populations of Caucasian/European ancestry, and the linked gene polymorphisms can also vary in different ethnicities. The relative contribution of Native American, African, and Caucasian/European ancestries in different countries—or even within the same country—may vary significantly. For instance, the Native American contribution is higher in Bolivia, Colombia, Venezuela, Peru, and Mexico, while the African contribution is higher in Brazil and coastal areas of Colombia and Venezuela; whereas in Argentina, the Caucasian/European contribution is higher than in the other countries. Gene polymorphism frequencies may be very different among the different ethnicities -for instance, a given polymorphism may be very common in Caucasian ancestry while being rare in Native American or African ancestry. In addition, the gene polymorphism frequencies also vary within the Native American and African populations themselves. This can lead to the failure to replicate an association in a different cohort. An additional problem is that several of the performed studies have small sample sizes for each disease category, which can either lead to failure to detect an existing gene polymorphism-disease association or, less frequently, to false positives due to sample bias. Small sample sizes may underlie the smaller number of gene polymorphisms associated with CCC severity, as well as with digestive/cardiodigestive cases, which are less frequent. Studies comparing gene polymorphism frequencies between two groups of *T. cruzi* seropositive Chagas disease patients (for instance, between ASY IF and CCC) tend to be more trustworthy than studies comparing *T. cruzi*-infected vs. noninfected subjects. This is because all Chagas disease patients are seropositive to *T. cruzi*, a proof of exposure to infection, and the gene polymorphism may be associated with differential disease progression. On the other hand, even when the seronegative controls come from the same endemic area and share the same ethnicity of Chagas disease patients, it is impossible to ascertain that all uninfected subjects have been exposed to the parasite and cleared the infection.

#### 2.1.1. Cardiovascular Gene Polymorphisms

Polymorphisms in cardiovascular genes have been previously implicated in other cardiac diseases and are associated with heart cell function and target organ pathophysiology. The rs640249 SNP, located in the cardiac actin *ACTC1* gene, is associated with CCC [50]. Microsatellite repeat polymorphisms in the cardiac myosin beta chain gene (*MYH7*) were not associated with CCC development [52]. The rs1805124 in the *SCN5A* gene, encoding the voltage-gated sodium channel alpha subunit 5 important for the initial upstroke in the cardiomyocyte action potential, is associated with CCC development [53]. The angiotensin-converting enzyme (ACE) gene D polymorphism, which is associated with increased angiotensin levels, is also associated with ischemic heart disease and has been found to be associated with CCC severity in a Brazilian study [54]. However, this association was not confirmed in two other Brazilian studies [55, 56].

#### 2.1.2. HLA Gene Polymorphisms

Regarding *HLA* class I and class II genes, which play a key role in T-cell recognition of parasite and host peptide antigens and are extremely polymorphic, several studies disclosed associations of different HLA class I and class II polymorphisms with CCC development [91, 94–100], and some studies showed negative results [52]. The lack of concordance of HLA association studies with Chagas disease may be due to differences in the genetic backgrounds of the studied cohorts.

#### 2.1.3. Cytokine and Cytokine Receptor Gene Variants

##### 2.1.3.1. Polymorphisms in Cytokine Genes Affecting IFN-*γ* Production and Th1 T-Cell Differentiation

Polymorphisms in genes affecting IFN-*γ* production and Th1 T-cell differentiation, like *IFNG*, *IL4*, *IL4RA*, *IL10*, *IL12*, *IL18*, and *EBI3/IL27B*, have been associated with susceptibility to *T. cruzi* infection, CCC, and CCC severity. *T. cruzi*-infected *il12b* knockout mice display increased parasitism and mortality [131], IL-12 being fundamental for the control of *T. cruzi* infection. *IL12B* SNPs, +1188 [77], rs2546893, and rs919766 [51], were shown to be associated with CCC development. IFN-*γ* is essential for the survival of *T. cruzi*-infected mice [132]. Furthermore, *IFNG* rs2430561 has shown an association with the acquisition of *T. cruzi* infection in a Colombian cohort [69], but this was not replicated in a Brazilian cohort [66]. This might reflect different ethnical compositions and the comparison of unequal samples—*T. cruzi* seropositive and seronegative, as discussed above.

*IL4* -590T (rs2243250) was associated with protection from infection in a Bolivian cohort [72], but this was not observed in a Colombian cohort [73]. The *IL4RA* + 148 AA genotype has been associated with CCC development in a Colombian cohort [73].

*T. cruzi* infection of *il10* knockout mice leads to increased levels of IFN-*γ* and proinflammatory cytokines, an intense myocarditis and lower survival [133], while higher IL-10 levels are associated with the IF of Chagas disease [12]. *IL10* SNPs −1082 rs1800896 [75], rs3024496 [51], −819 rs1800871, −592 rs1800872 [76] were associated with the development of CCC; the association of -819 rs1800871 to CCC was sustained in a meta-analysis—a study made on the pooled data from several studies—with 754 CCC cases and 385 controls from Argentinian, Brazilian, and Colombian cohorts [76]. EBI3/IL-27B dampens IFN-*γ* driven inflammation by inducing IL-10-producing Tr1 cells, and plasma levels of EBI3/IL-27B are higher in indeterminate/ASY Chagas disease patients than CCC patients. Certain genotypes of the rs4740 and rs4905 polymorphisms in the *EBI/IL27B* gene were associated with protection against CCC development [31].

*Il18r1* knockout mice display lower levels of Th1 cells and are highly susceptible to *T.cruzi* infection [134], and IL-18 induces cardiac remodeling and cardiomyocyte hypertrophy [135]; furthermore, *IL18* expression is upregulated in CCC heart tissue [26, 28]. Some *IL18* variants were associated with susceptibility to *T. cruzi* infection in Colombian cohorts [81, 82]; the *IL18* promoter functional polymorphism rs360719 was found to be associated with susceptibility to *T. cruzi* infection in a Colombian cohort [81]. The functional variant rs2043055, which is located in an enhancer region of *IL18* expression, was associated with infection in two Colombian cohorts [81, 82] and with CCC severity in a Brazilian cohort with 1051 patients [83]. A meta-analysis based on data from 3608 patients from Colombia, Bolivia, Argentina, and Brazil, the rs360719 variant of *IL18* replicated the association with susceptibility to *T. cruzi* infection, while the association of rs2043055 with CCC was not replicated [81].

##### 2.1.3.2. Polymorphisms in Other Cytokine Genes

IL-17A is important for the resolution of *T. cruzi* infection in mice [136] and is higher among ASY Chagas disease than CCC patients [15]. The variant rs8193036, located in the *IL17A* gene, was associated with infection and CCC development in a Colombian cohort [78], while *IL17A* rs763780 was associated with *T. cruzi* infection in a Brazilian cohort. A meta-analysis involving 2967 individuals in Colombia, Argentina, Bolivia, and Brazil indicated that *IL17A* rs4711998 was associated with infection [80]. RS2275913 in the *IL17F* gene has been associated with CCC severity [79]. The *TNFA—*308 gene variant previously linked to increased TNF-*α* production—was associated with CCC development in a meta-analysis of Mexican, Peruvian, and Brazilian cohorts [4].

The +5810G/A and +5810G/G polymorphisms in the *IL1B* gene were associated with CCC development [70]. IL1R is the receptor for IL-1. The gene polymorphism *IL1RN.4* was associated with *T. cruzi* infection [71]. TGF*β*1 facilitates *T. cruzi* entry and replication within host cells, as well as promoting fibrosis and modulating inflammation [137]. *TGFB1* gene polymorphisms, −509 rs1800469, and +10 rs1800470 were associated with susceptibility to infection in a Brazilian cohort [67]; the association with +10 rs1800470 was replicated in cohorts from Peru and Colombia [68]; however, the association with −509 rs1800469 was not replicated in a Brazilian cohort [66] and in a Colombian/Peruvian cohort [68]. MIF is a proinflammatory cytokine, and the functional promoter polymorphism-173 is associated with susceptibility to infection [84].

#### 2.1.4. Chemokine and Chemokine Receptor Polymorphisms

Chemokines and chemokine receptors play a major role in *T. cruzi* infection and CCC pathogenesis since chemokines attract chemokine receptor-positive inflammatory cells into infected and/or inflamed tissues like the heart. CCL2 is a chemokine-attracting monocytes that express its receptor CCR2 into inflamed tissues; both CCL2/MCP-1 and CCR2 expression is upregulated in hearts of CCC patients [27]. Two *CCL2* SNPs were associated with CCC development: SNPs-2518, rs1024611, [57] and rs2530797, which is in strong linkage disequilibrium with rs1024611 [49]. CCL5 attracts inflammatory cells, including Th1 T cells expressing its receptor CCR5 to inflamed tissues and is the most upregulated chemokine in CCC heart tissue. The *CCL5*-403 (rs2107538) promoter polymorphism has been shown to confer protection against CCC [58].

Chemokines CXCL9 and CXCL10, which attract CXCR3+ Th1 T cells, show increased expression levels in the hearts of CCC patients [28, 29]. Genotypes of SNPs *CXCL10* (rs3921) and *CXCL9* (rs10336) are associated with CCC severity. Significantly, the same *CXCL9* (rs10336) genotype was also associated with cardiac expression of CXCL9 mRNA and intensity of myocarditis in the hearts of CCC patients, indicating this is a functional variant that regulates gene expression and myocarditis intensity [29].

Chemokine receptor polymorphisms may also be involved in CCC severity, as evidenced by the variant rs1799864 located in the *CCR2* gene [60]. CCR5 is a chemokine receptor found in inflammatory Th1 T cells and binds the chemokines CCL3, CCL4, and CCL5, whose mRNAs are highly expressed in the hearts of CCC patients, particularly *CCL5* [29]. *CCR5* polymorphisms have been extensively studied in Chagas disease and associated with distinct disease forms in different cohorts. *CCR5* gene variants such as rs1800024 [60] and −2554T and rs2734648 [59] and rs1799988 [29] were associated with CCC severity in different cohorts. The *CCR5* rs1799987 polymorphism is associated with CCC [61] in Peru and the digestive form in Brazil [63]. However, these associations were not confirmed in Colombian [59, 60], Venezuelan [62], and Brazilian [58] CCC cohorts. Conversely, *CCR5* rs3176763 provides protection against CCC [49]. Additionally, a study identified human haplogroup (HH)-A of the CCR2-CCR5 loci as associated with CCC [60]. The 32 bp deletion in *CCR5* (Δ32 rs333), which is associated with reduced receptor activity, showed no associations in the Peruvian [61], Venezuelan [62], and Brazilian [63] cohorts. A meta-analysis of Colombian, Venezuelan, Peruvian, Bolivian, Argentinian, and three Brazilian studies on the association of CCR5 polymorphisms with CCC [138] disclosed that CCR5 rs2856758 and rs2734648 are associated with CCC development; when the analysis was limited to patients from countries originating from Spanish colonization (predominant European/Caucasian and Native American ancestry; excluding Brazil, with more African and less Native American ancestry), CCR5 rs1799987, rs1799988, rs1800024, rs1800023 were found to be associated with CCC.

#### 2.1.5. Polymorphisms in Other Innate Immunity Genes

TLR4 signaling is important for protection against *T. cruzi* infection. The *TLR4* haplotype D229G/T399I has been associated with protection from CCC in a Chilean cohort [116], which was replicated in a Venezuelan cohort [125] and also associated with *T. cruzi* infection [125]. However, the D299G polymorphism was not associated with disease in a Brazilian [118] and a Colombian cohort [124]. TIRAP is an important adaptor protein in the intracellular signaling pathway of TLRs, and both the missense variant S180L (rs8177374) and rs8177376, which is in strong linkage disequilibrium with rs8177374, are associated with CCC development [50, 118] indicating replication of the association. However, the S180L variant was not associated with disease in a Chilean cohort [116].

NFKBIL1 contributes to the negative regulation of TLR and interferon signaling pathways by acting on transcriptional activation of NF-*κ*B target genes in response to endogenous proinflammatory stimuli. The *NFKBIL1* promoter polymorphism-324 is associated with CCC development [122]. *DDX39B/BAT1* is a gene encoded in the HLA region, 40 KB away from the *TNFA* locus, and can downmodulate TNF-*α* and IL-6 production. The *BAT1*-22 and -348 promoter polymorphisms, associated with reduced expression of BAT1, were associated with CCC development in a Brazilian cohort [130]. Canonical PI3KG signaling in myeloid cells is essential to restrict *T. cruzi* heart parasitism, myocarditis, and death of mice during acute infection, and PI3KG mRNA expression inversely correlates with parasitism in the hearts of CCC patients [139]. The rs1129293 *PIK3CG* polymorphism is associated with PIK3CG expression and CCC development [127].

Inflammasome activation is important for the control of *T. cruzi* infection [140]. The *NLRP1* rs11651270 polymorphism is associated with CCC development [106]. Caspase 1 (CAS1) activation prompts inflammatory cytokine secretion. The *CAS1* SNP rs501192 is associated with a trend to CCC development [128].

Complement is important for protection against *T. cruzi* infection, and polymorphisms in genes encoding complement system components affect infection and symptomatic disease development. The *C3F* variant is associated with CCC development, while the *BFS* variant is associated with protection from infection [129]. The complement receptor 1 polymorphisms *CR1* rs1704660G, rs17047661G, and rs6691117G are associated with acquisition of T. cruzi infection, while the *CR1⁣*^*∗*^AGGGTG is associated with CCC development [108].

Collectin-11 and MASP2 are involved in complement activation. Collectin-11 binds to mannose and fructose in microorganisms and activates complement after interaction with MASP2, which cleaves complement components C2 and C4. Collectin-11 plasma levels were reduced in Chagas disease patients. Certain genotypes of *COLEC11* in rs7567833, rs7567833, and rs7567833 and the *COLEC11⁣*^*∗*^GGC haplotype were associated with *T. cruzi* infection and progression to CCC and cardio-digestive forms of Chagas disease [107]. Several polymorphisms of the MASP2 gene are associated with CCC development [117]. *MASP2* rs1961795 is associated with the development of CCC and the digestive forms [107]. *COLEC11* rs7567833G and *MASP2* Chagas disease risk genotypes may act synergistically to increase the risk of developing CCC Chagas disease [107]. Ficolin 2 is a lectin binding to carbohydrates that activates the lectin complement pathway, and the rs17514136 located in the *FCN2* gene is associated with CCC development [111].

NK cells are activated early in acute *T. cruzi* infection, and their IFN-*γ* production is important for parasite control. Killer cell immunoglobulin-like receptors (KIRs) are transmembrane glycoproteins expressed by NK cells and subsets of T cells. *KIR2DS2-//KIR2DL2-/Haplotype KIR2DL3+/C1* are associated with digestive form [115], while *KIR2DS2+/KIRD2L2-/HLA-C1* is associated with CCC and CCC severity [119]. Significantly, KIR2DS2 is an NK-activating KIR, while KIR2DL3 is an NK-inhibiting KIR. MICA molecules bind to the activating NKG2D receptor in NK cells. The *MICA-129* (rs1051792) genotype met/met was associated with CCC severity [119, 120] and digestive forms of Chagas disease [115]. The haplotype *MICA*⁣*^*∗*^008~HLA-C^*⁣*^*∗*^^06* was associated with the digestive form of the disease [115].

#### 2.1.6. Extracellular Matrix Remodeling Gene Polymorphisms

Extracellular matrix remodeling gene polymorphisms play a significant role in susceptibility to congenital *T. cruzi* infection. ADAM12 and MMP2 play a role in extracellular matrix remodeling. A study conducted by Juiz et al. [93] in 2016 identified two polymorphisms, rs11244787 and rs1871054, located in the *ADAM12* gene, as well as three SNPs (rs243866, rs17859821, and rs2285053) in the *MMP2* gene, that are associated with congenital infection. These findings suggest that alterations in genes responsible for extracellular matrix remodeling may influence host susceptibility to congenital transmission of *T. cruzi*, potentially facilitating parasite transmission across the placenta. These SNPs provide crucial insights into the genetic mechanisms underlying congenital transmission and offer a basis for future research into potential therapeutic targets or genetic markers for predicting disease susceptibility.

#### 2.1.7. Other Genes

VIP is a neurotransmitter with anti-inflammatory properties, and lower levels of VIP were found in CCC than in indeterminate Chagas disease patients; *VIPR1* and *VIPR2* encode VIP receptors, and *VIPR1* SNP rs342511 and *VIPR2* SNP rs885861 were associated with protection from CCC and CCC development, respectively [102]. The CTLA-4 is an immune system inhibitory molecule. *CTLA4* variant rs733618 was associated with protection to CCC, and rs5742909 was associated with the cardio-digestive form [109]. Haptoglobin (HP) is an acute phase response protein produced in the liver in response to inflammatory cytokines and other inflammatory stimuli; it binds free plasma hemoglobin and has antioxidant properties. HP2 has been shown to be associated with CCC severity [104]. The HP1 allele and the HP1-1 genotype were associated with protection against CCC development [104, 105].

#### 2.1.8. Polymorphisms Associated With Severe CCC

Most patients who die from Chagas disease are severe CCC patients who have significant ventricular dysfunction (left ventricular EF ≤ 40%); moreover, their mortality is significantly higher than that of noninflammatory etiologies of cardiomyopathy. Therefore, polymorphisms associated with the development of severe CCC as compared to mild CCC (left ventricular EF >40%) have a special pathophysiological and prognostic interest. These polymorphisms are found in genes (1) involved in the differentiation of monocytes and IFN-*γ*-producing Th1 T cells to the heart tissue of CCC patients, like *IL18* (Th1 differentiation), *IL27B/EBI3*, associated with negative modulation of IFN-*γ* responses and higher in plasma of the indeterminate/ASY form of Chagas disease; (2) chemokine and chemokine receptor polymorphisms (*CXCL9/Mig* and *CXCL10/IP10*, *CCR2*, *CCR5*) involved in Th1/monocyte migration; (3) *IL17F*, associated with the IL-17 response; (4) innate immunity genes such as KIR and MICA, involved in activation/inhibition of NK cells, which play an important role restricting *T. cruzi* growth in acute infection via IFN-γ production and direct cytotoxicity, and which are less activated in severe than moderate CCC [33]; (5) CASP1, important for innate immunity receptor/inflammasome induced maturation of IL1 beta and IL-18 secretion, as well as pyroptosis; (6) HP, an acute phase response protein produced in the liver in response to cytokines and other inflammatory stimuli, which has antioxidant, anti-inflammatory and immunomodulatory properties [141]; and (7) ACE, the ACE involved in renin–angiotensin–aldosterone system (RAAS) modulation that is important in heart failure observed in CCC. Overall, they suggest that control of *T. cruzi* infection, differentiation and migration of Th1 T cells and monocytes, NK cells, oxidative status, and RAAS modulation might be primary drivers of the transition from low-to-high mortality CCC. One caveat of this analysis is that, in many studies, even when sample sizes of the CCC vs. the ASY/IF were adequately powered, subdivision of CCC in moderate vs. severe CCC made sample sizes too small to detect significant differences among them.

#### 2.1.9. Summary of Common Gene Polymorphisms in Disease Progression

**Figure 1:**
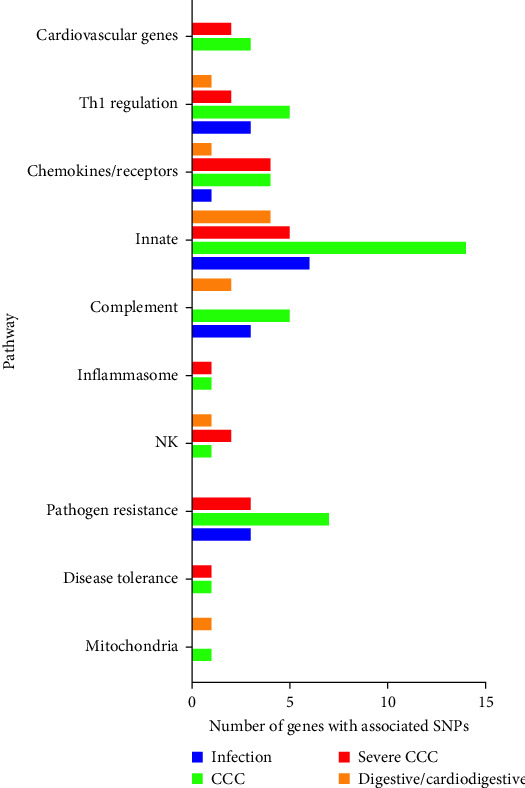
Immune pathways of genes encoding SNPs associated with progression to distinct clinical forms of Chagas disease.

**Figure 2:**
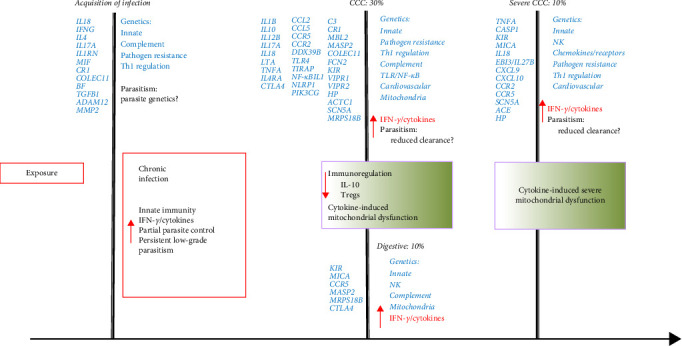
Gene polymorphisms and checkpoints for the acquisition of infection and development of clinical forms of Chagas disease.

In Figures 1 and 2, we attempt to summarize the results of case–control studies of common gene polymorphisms and put in perspective the pathophysiological pathways these genes belong to. In Figure 1, we plotted the number of variant genes associated with infection, CCC, severe CCC, and digestive disease per pathophysiological pathway. In Figure 2, we display the genes with polymorphisms significantly associated with each checkpoint for Chagas disease progression, as well as the immunopathological/pathophysiological pathways to which these genes belong to. This way, we observe that polymorphisms in genes from the innate immunity and pathogen (*T. cruzi*) resistance pathways are important for susceptibility to infection, CCC, severe CCC, and digestive/cardiodigestive disease, suggesting that control of parasite burden may be involved in susceptibility to all clinical forms. A breakdown in the innate immunity pathway shows that polymorphisms in genes from the complement pathway are important for susceptibility to infection and digestive/cardiodigestive forms, indicating complement-dependent clearance of *T. cruzi* is important for the acquisition of infection and progression to digestive forms. TLR/NF-*κ*B and inflammasome pathway polymorphisms are important for progression to CCC, while polymorphisms in genes linked to NK cells are important for digestive disease and severe CCC. Polymorphisms in genes from the Th1 regulation pathway are important for the acquisition of infection, susceptibility to CCC, and severe CCC. Polymorphisms in genes from the chemokines/receptors pathway are particularly important for susceptibility to CCC and severe CCC, implying that control of migration of T cells and monocytes to the heart is especially important for progression to CCC and severe disease. Polymorphisms in extracellular matrix genes are relevant to the acquisition of infection. Polymorphisms in cardiovascular genes are important for susceptibility to CCC and severe CCC. The limitation of this display is that many polymorphisms were not tested against all clinical forms, and several of the associated gene polymorphisms were observed in only one country cohort; a limited number of polymorphisms was tested in different cohorts, and the association may not have been replicated in different cohorts or in meta-analyses of different cohorts.

##### 2.1.9.1. Replication of Gene Polymorphism Associations and Meta-Analysis in Disease Progression

Independent replication of gene polymorphism-disease association in distinct cohorts reinforces the credibility of the association. Confirmation of gene polymorphism-disease associations in meta-analysis of multiple studies also increases the credibility of associations. Taking into account all studies reported in this review, independent replication or confirmation of gene polymorphism-disease association in meta-analyses were positive for 15 polymorphisms in 10 genes, as follows: *CCL2* rs1024611 [57] and rs2530797 with CCC (strong linkage disequilibrium) [49]; *CCR5* rs2856758 and rs2734648 and CCC [138]; *CCR5* rs1799987, rs1799988, rs1800024, rs1800023 were also associated with CCC in meta-analyses of cohorts from countries originating from Spanish colonization [138]. *LTA* -+252 and CCC [64, 65], *TNFA*-308 and CCC [4], *IL10*-819 rs1800871 and CCC [76], *TLR4* haplotype D229G/T399I with protection from CCC [116, 125], *TIRAP* S180L (rs8177374) and rs8177376 (strong linkage disequilibrium) with CCC [50, 119], *IL18* rs360719 and infection [81], *IL17A* rs4711998 and infection [79], *TGFB1*+10 rs1800470 with infection [67, 68]. Among the polymorphisms associated in one cohort which were tested in different cohorts but where the association was not replicated, we have *IL18* rs2043055 with CCC [81, 82], *IFNG* rs2430561 and infection [66, 69], *IL4* −590 and infection [72, 73], *TGFB1* −509 rs1800469 and infection [66, 68].

#### 2.1.10. GWASs

Three studies have been specifically devoted to uncovering common gene variants associated with susceptibility of CCC development over the whole genome in an “agnostic” approach without candidate genes employing GWAS. In GWA studies, adjustment of *p* value for multiple comparisons set the genome-wide significance cutoff to 10^−8^. The first study, comparing 600 Brazilian CCC and ASY patients, identified no variants significantly associated with pathology [142]. Nevertheless, 46 variants showed patterns of association with phenotypic traits of interest, including EF, PR, QRS, QT intervals, antibody levels by EIA, and parasitemia by polymerase chain reaction. Interestingly, most of these mutations are located within genes linked to CCC or to biological processes dysregulated in CCC. The second study performed a meta-analysis, combining several GWAS analyses. It was carried out on a larger cohort, including data from Brazilian patients with 1796 seropositive (1022 ASY, 774 CCC) and 1104 seronegative patients from three different countries (Colombia, Argentina, and Bolivia). Although no mutation has been associated with Chagas disease, a variant located near the *SAC3D1* gene, involved in the regulation of the immune response, appears to be involved in the development of cardiac forms [105]. Moreover, computational analysis indicates that this variant may exert an influence on the *SNX15*, *BAFT2*, and *FERMT3* genes, three genes associated with cardiovascular traits. The latest study was restricted to the Brazilian population and included 2964 patients (581 ASY, 2383 CCC). It identified a variant associated with the immune response located in the *AKAIN1* gene (C18orf42) [143].

#### 2.1.11. ChagasDB: A Database of Molecules Involved With *T. cruzi* Infection and Chagas Disease

The public database ChagasDB (https://chagasdb.tagc.univ-amu.fr/) [144] is a searchable, manually curated database that gathers all the molecules associated with *T. cruzi* infection in different host species, including genes, proteins, polymorphisms, hormones, or other chemical compounds. It reveals that there are 57 proteins functionally linked to the 68 susceptibility loci associated with immune response, including inflammation and Th 1 response. While these findings may not distinctly emphasize certain mutations of interest, they do, however, validate known variants previously associated with CCC, such as *IL18* [81] or cardiac hypertrophy, as seen with *HSPB8* [145]. The presence of susceptibility loci within genes or their association with genes connected to dysregulated functions in CCC suggests their potential involvement in the pathogenicity of CCC.

#### 2.1.12. Rare and Intermediate-Frequency Pathogenic Missense/Nonsense Variants in Nuclear-Encoded Mitochondrial and Inflammatory Genes Variants Are Associated/Cosegregate With Symptomatic Forms of Chagas Disease: Functional Implications

The use of high-throughput next-generation sequencing technologies was able to agnostically identify variants in new genes participating in pathobiological pathways important for CCC. In Chagas disease, two studies adopted the whole exome sequencing approach and identified variants in several genes belonging to different pathobiological relevant to disease pathogenesis. The first study was performed on six nuclear families, with multiple cases of the cardiac form recruited in the endemic regions of Bahia and Minas Gerais states, Brazil [146]. In each family, the study searched for rare missense/nonsense pathogenic variants of genes shared by all family members with CCC but absent in infected ASY/IF siblings and unrelated ASY/IF subjects, that were part of patho-biologically relevant processes in CCC, like inflammation, fibrosis, mitochondria, arrhythmia, oxidative stress, etc. [146]. This study identified heterozygous pathogenic rare variants linked to CCC in all families tested on 22 distinct genes, 20 of which were either mitochondrial or inflammation-related; mitochondrial variants segregated with CCC in five out of the six studied families, and inflammation-related genes segregated with CCC in five of the six families.

Genetic disorders involving mitochondrial genes are the most common congenital genetic syndromes. Homozygous pathogenic variants of at least 250 nuclear-encoded mitochondrial genes, along with variants in mitochondrial DNA (mtDNA), are causative of mitochondrial genetic disorders [147]. Each leads to drastic mitochondrial dysfunction, energetic and functional impairment in multiple organs, in special heart and nervous tissue, which are the most avid consumers of adenosine 5′-triphosphate (ATP) in the body, although clinical penetrance is variable. Interestingly, up to 30%–50% of genetic mitochondrial disease patients develop cardiomyopathy, heart conduction defects, heart failure, ventricular arrhythmia or sudden cardiac death, and autonomic nervous system imbalance [148]. In addition, 15% of them develop gastrointestinal motility disorders due to denervation and destruction of myoenteric neurons—including megaesophagus and megacolon [149]. The striking similarity in the proportions of patients with cardiac, digestive, and autonomic disorders in mitochondriopathies and the clinical spectrum of symptomatic Chagas disease (30% progress to CCC, 10% to gastrointestinal disorders associated with local neuron loss) made us hypothesize that heterozygous pathogenic mitochondrial variants, which may cause a partial reduction in mitochondrial function, may play a role in differential progression for CCC. Among these mitochondrial gene variants segregating with CCC were dihydroorotate dehydrogenase (*DHODH*) R135C (rs201230446), which encodes an enzyme donating electrons to the electron transfer chain (ETC) and participating in pyrimidine metabolism; this variant had been previously reported as a complete loss of function mutation, and its presence was associated with mitochondrial dysfunction [150]. Incubation of AC16 cardiomyocytes with IFN-*γ* and TNF-*α* significantly reduced mitochondrial membrane potential (MMP) (Δ*ψ*M), and cotreatment with brequinar, a DHODH inhibitor, further reduced MMP to levels below those observed with cytokine treatment alone. This suggested that mitochondria from cardiomyocytes with defective DHODH function are more susceptible to cytokine-induced mitochondrial dysfunction than those with normal enzyme function [146].

**Figure 3:**
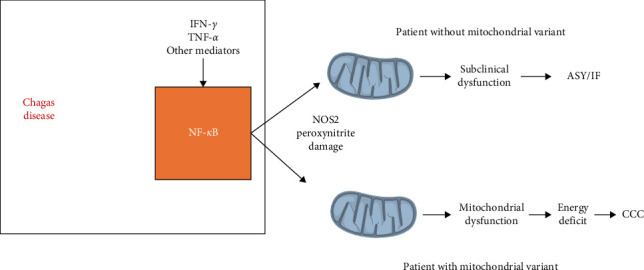
Pathogenic mitochondrial gene variants putatively increase susceptibility to cytokine-induced mitochondrial dysfunction in organs with inflammation.

In a different family, we found the S30G variant in the uridine monophosphate synthase gene (*UMPS;* rs17843776), the enzyme following DHODH in the pyrimidine biosynthesis pathway; UMPS has as substrate the orotate produced by DHODH, and a reduction of enzyme activity could cause accumulation of orotate, which could in theory inhibit DHODH activity. We also found a stopgain/nonsense variant W269X in the *RPUSD3* gene (rs142984515), which created a C-terminal deletion of 25% of the RPUSD3 protein product; RPUSD3 is important for the translation of mtDNA-encoded proteins that are part of the ETC. We also found the V120M variant in the *MRPSB18* gene (rs116524936), which encodes a protein that is part of the small 28S subunit of the mitochondrial ribosome [146]. These variants might induce disruption of translation and transcription of mtDNA-encoded genes participating in the ETC, which could reduce energy production and/or induce excessive oxidative stress. Indeed, preliminary studies with cells carrying the RPUSD3 truncation variant showed reductions in several ETC complexes. Since inflammatory cytokines such as IFN-*γ* and TNF-*α* by themselves induce mitochondrial dysfunction [151, 152], this study hypothesized that carriers of heterozygous mitochondrial gene variants might display increased susceptibility to cytokine-induced mitochondrial dysfunction in inflamed tissues like the heart, thus participating in the pathogenesis of CCC [146]. Preliminary unpublished results have shown that cardiomyocytes carrying such heterozygous mitochondrial mutations display reduced mtDNA content and are, in general, more susceptible to cytokine (IFN-*γ* and TNF-*α*) induced mitochondrial dysfunction than wild-type cells (Figure 3). This mechanism, whereby pathogenic variants in mitochondrial genes synergize with inflammatory cytokines produced locally lead to significant mitochondrial dysfunction, may be operative in other kinds of heart disease with significant inflammatory cytokine involvement, like viral myocarditis, inflammatory cardiomyopathy, cancer chemotherapy-induced cardiac toxicity, and cardiac aging-associated heart dysfunction [153].

A second whole exome sequencing study was carried out on patients with the digestive form of Chagas disease, more specifically, the Chagasic megaesophagus. This study involved whole exome sequencing of unrelated patients with Chagasic megaesophagus and unrelated ASY/indeterminate Chagas disease patients. In this study design, we looked for intermediate frequency/common variants. A missense pathogenic variant in the same *MRPSB18* gene found in the family study, but in a different position (P230A, rs34315095) was found to be more frequently associated with patients with megaesophagus (38%) than with ASY/indeterminate patients (2.7%; *p*=0.015, OR = 11, 95% CI [1, 6, 56–128]) [101]. The variant also occurs in 18% of CCC patients and has a frequency of 2% in the Brazilian whole genome and exome sequence database ABRAOM (https://abraom.ib.usp.br/). We performed a functional assessment of the *MPRS18B* P230A variant in Epstein–Barr virus-immortalized lymphoblastoid cell lines (LCLs) derived from the blood of megaesophagus patients carrying or not the *MRPSB18* 688C >G, P230A variant. After treatment with IFN-*γ*, LCL from patients carrying the heterozygous mutation showed increased nitro-oxidative stress as compared to those not carrying the variant. In addition, an LCL line homozygous for the *MRPSB18* P230A variant also showed an increase in IFN-*γ* induced mitochondrial superoxide and reduced levels of ATP production as compared to cells from patients with the (C/C) genotype. The authors hypothesized that in megaesophagus patients carrying the mitochondrial variant *MRPSB18* P230A variant, the locally increased inflammatory cytokines produced by the inflammatory infiltrate in the esophageal myenteric plexus could induce nitrooxidative stress and mitochondrial dysfunction leading to the death of neurons, contributing to the denervation observed in Chagasic megaesophagus [101]. These results support the hypothesis that pathogenic mitochondrial mutations may synergize and potentiate nitro-oxidative stress and mitochondrial dysfunction in inflammatory settings not only in the heart but also in other energy-demanding tissues and organs. Unpublished preliminary studies on exome sequencing in unrelated CCC patients identified additional pathogenic mitochondrial variants associated with CCC with low to intermediate frequencies in the Brazilian population.

#### 2.1.13. Mitochondrial Haplotype and Disease-Associated mtDNA-Encoded Variants Are Associated With Symptomatic Forms of Chagas Disease

A study conducted by Gallardo et al. [154] in 2023 investigated mtDNA variants and employed high-throughput sequencing (Hi-SNPseq) on cardiac tissue from both healthy individuals and patients with CCC, as well as in blood from Chagas disease patients in the ASY, moderate and severe CCC clinical forms. This analysis revealed the distribution of mitochondrial haplogroups in Chagas disease and found that the European macro-haplogroup H, previously associated with increased oxygen consumption and mitochondrial oxidative damage [155], was associated with an elevated risk of CCC [154]. Moreover, the study identified genetic variants that were more prevalent in CCC patients, based on heteroplasmy, with 712 variants significantly associated with distinct disease forms (ASY, moderate CCC, or severe CCC) [154]. Notably, 70 of them were previously associated with mitochondrial genetic syndromes, 40 of them being pathogenic missense variants in mtDNA-encoded proteins; four of these variants are already implicated in heart disease [154]. mtDNA disease variants were found to increase the expressivity and severity of cardiomyopathy associated with nuclear-encoded mitochondrial gene variants [156], implying a synergy between the two variant types. These findings suggest that variants in nuclear-encoded mitochondrial proteins and mtDNA variants may play a role in the genetic predisposition to CCC.

## 3. Mitochondria and Chagas Disease Cardiomyopathy

As mitochondrial function and quality control influence the development of multiple diseases, including cardiovascular disorders and heart failure of different etiologies [157], over the past two decades, several studies have explored the impact of mitochondrial impairment in the heart on chronic Chagas disease both in murine models [158] and CCC patients [159]. Earlier data from murine models of CCC indicate that chronic infection impacts cardiac energy production primarily through mitochondrial dysfunction, disrupting energy metabolism pathways [160, 161]. Mitochondrial defects of complex III and V (ATP synthase) of the ETC were found in the heart of chronically *T. cruzi*-infected mice, along with a substantial decline in cardiac mtDNA content and mitochondria-encoded transcripts [160]. This indicates that mtDNA alterations contribute to the deficiencies in oxidative phosphorylation (OXPHOS) activity in murine models of CCC, leading to an increase in ROS release and reduced ATP production [160]. Excessive ROS can be harmful to several cellular molecules, including those within the mitochondria. Elevated oxidative markers, including hydrogen peroxide (H_2_O_2_) carbonyls and malondialdehyde (MDA), along with mitochondria oxidative damage markers, have been observed in the myocardium of chronically *T. cruzi*-infected mice [162]. The antioxidant system may be compromised in the chronic stage due to a decrease in manganese superoxidase dismutase (MnSOD) activity and a decline in the total antioxidant capacity (TAC) in cardiac mitochondria from *T. cruzi*-infected mice [162–164].

Cardiomyocytes are specialized muscle cells that form the myocardium. They are responsible for the constant contraction and relaxation of the heart, which requires a constant flow of energy [165, 166]. Cardiomyocytes obtain energy by oxidation of various energy sources, including fatty acids (FAs), glucose, and ketone bodies, depending on the physiological conditions [165, 166]. Energy metabolism of cardiomyocytes relies on the proper function of mitochondria and consists of three main components: oxidation of FAs, the primary energy source, responsible for up to 90% of the ATP consumption; the TCA, which generates substrates for OXPHOS; glycolysis, which provides pyruvate and Acetyl-CoA for the TCA and is responsible for 10%–25% of the total energy production of the myocardium; and the creatine kinase system, which delivers mitochondrial ATP to cytoplasm and sarcomeres, in situations of high demand [166–172]. CCC has a worse prognosis than cardiomyopathies of noninflammatory etiology. In human CCC, myocardial mtDNA content is significantly reduced as compared with samples from idiopathic dilated cardiomyopathy (DCM) or control heart tissue, with increased nitro-oxidative stress [161], MDA [165], and 4-hydroxynonenal levels [173]. A reduction of high-energy phosphates, indicative of reduced ATP production, has been observed in vivo in the hearts of CCC patients, that was already present in patients without ventricular dysfunction, but was more intense in the presence of ventricular dysfunction [174]. A proteomic study by our group disclosed impairment of multiple mitochondrial energy metabolism pathways (reduced expression of FA oxidation, TCA cycle, OXPHOS, creatine shuttle enzymes) in the heart of CCC patients, which in many cases was more intense than other, noninflammatory cardiomyopathies such as DCM and IC [175]. Importantly, protein levels of six FA beta-oxidation enzymes were found to be reduced in CCC heart tissue, as compared to 1 and 2 in IC and DCM, respectively, consistent with a reduced function of the FA beta-oxidation pathway more intense in CCC heart tissue. This was corroborated by the metabolomics profiling of CCC heart tissue, showing significantly decreased levels of myocardial acylcarnitines and long-chain FAs as compared to healthy controls [176]. Conversely, five glycolytic enzymes were increased in CCC heart tissue, as opposed with three in DCM, and upregulation of OXCT1, the key enzyme for ketone body catabolism. Altogether, results indicate a shift away from FA oxidation and toward glycolysis and ketone body metabolism. This shift in energy sources is common in heart failure but is especially intense in CCC. Together with data from high energy phosphates dysfunction [174], this energetic failure may explain the worse prognosis of CCC. A reduction in the protein expression of the mitochondrial antioxidant peroxyredoxins, all present in mitochondria, and an increase in catalase levels was observed in CCC heart tissue, are consistent with reduction of antioxidant responses and increased nitro-oxidative stress, in line with above mentioned increases in malonaldialdehyde [165], 4-hydroxynonenal protein adduct levels [173] and 3-nitrotyrosine-modified proteins [161].

### 3.1. Cytokine-Induced Mitochondrial Dysfunction in Chagas Disease Cardiomyopathy

*T. cruzi*-infected cells are absent from most areas of inflamed heart tissue in CCC patients, and thus, *T. cruzi* infection in the heart per se is not the likely driver of altered cardiomyocyte function in human CCC [177]. On the other hand, inflammatory cytokines like IFN-*γ* and TNF-*α* are abundant in CCC myocardium [11, 24, 25]. IFN-*γ* and TNF-*α* are the top upstream regulators of the transcriptional profile of CCC myocardium [28], and a strong IFN-*γ* transcriptional signature has been found in CCC heart tissue [27, 28]. Taken together, this indicates that IFN-*γ* and TNF-*α* are the key cytokines modulating transcriptional changes in CCC myocardium. Such cytokines are known to induce mitochondrial dysfunction in vitro [151, 152, 178, 179], and transgenic mice overexpressing IFN-*γ* in plasma develop a TNF-*α* dependent myocarditis and cardiomyopathy [30], which confirm IFN-*γ* is the culprit in CCC.

IFN-*γ*/TNF-*α* driven NF-*κ*B activation causes impairment of the MMP and ATP synthesis, mainly through inducible nitric oxide synthase (iNOS) induction and increase in NO/ROS/peroxynitrite levels [151, 178]. In addition, TNF-*α*-induced reduction in the activity of ETC complex I [152, 179] as well as that of master regulators of mitochondrial energy metabolism, like adenosine monophosphate-activated protein kinase (AMPK), peroxisome proliferator–activated receptor gamma coactivator-1Alpha (PGC-1*α*), and carnitine palmitoyl transferase I (CPT-1) [180], which contribute to reduced ATP production.

In vitro treatment of cardiomyocytes with STAT1, NF-*κ*B, iNOS, and p38 MAPK antagonists, as well as AMPK, SIRT1, or NRF2 agonists, mitigated IFN-*γ*/TNF-*α* -induced reduction in MMP [161]. This is in line with the finding that in vivo treatment with AMPK, SIRT1, or NRF2 agonists in a murine model of CCC led to the reduction of cardiac inflammation, oxidative stress, and improvement of ventricular function, at least in part via antagonism of NF-*κ*B activation [181].

Our group has shown that treatment of the human cardiomyocyte cell line AC16 with inflammatory cytokines IFN-*γ* and TNF-*α* for 48 h leads to decreased mtDNA, increased levels of nitrosative and oxidative stress, increased electron leak from ETC, decreased MMP, decreased mitochondrial and glycolytic ATP production, and reduced dependency on FA beta-oxidation [161].

Proteomic analysis indicated that cytokine treatment of AC16 cardiomyocytes led to reduced expression of proteins involved in FA oxidation, TCA, OXPHOS, and antioxidant response and increased expression of proteins involved with glycolysis and mitochondrial biogenesis that were similarly altered in CCC heart tissue. Four-week IFN-*γ* treatment of human cardiomyocytes derived from human-induced-pluripotent stem cell-derived cardiomyocytes (hIPSC-CM) led to a reduction of both glycolytic and OXPHOS activity, indicating that chronic IFN-*γ* stimulation mediates metabolic changes in cardiomyocytes compatible with the transcriptomic profile observed in aging myocardial cells, as well as in failing hearts [153].  Table 2 shows cytokine-induced changes and pathways analysis of proteomic and transcriptomic profiling of CCC heart tissue and cytokine-treated AC16 cardiomyocytes, disclosing the similarities in inflammatory and mitochondrial/energy metabolism in both situations.

Taken together, these findings suggest that treatment of cardiomyocytes with IFN-*γ* and TNF-*α* recapitulates the mitochondrial and metabolic dysfunction observed in CCC heart tissue, thus suggesting that cytokine-induced mitochondrial dysfunction is a key pathogenic factor and therapeutic target. Mitochondrial damage triggered by various stimuli can lead to inflammation. ROS activate NF-*κ*B, while dysfunctional mitochondria release mitochondrial DAMPs (damage-associated molecular patterns), including mtDNA, into the cytoplasm. This release activates several key inflammatory pathways, including TLR9/Myd88/NF-*κ*B, cGas/STING, Type I IFN, and the NRLP3 inflammasome. This mechanism has been implicated in sterile cardiac inflammation, which occurs in heart failure across diverse etiologies [182]. Mitochondrial dysfunction-induced sterile inflammation may play a critical role in sustaining inflammation in the absence of the parasite, potentially exacerbating the inflammatory processes seen in CCC.

Considering the aforementioned findings, the inflammatory environment initiated by the parasite sets the stage for a scenario where cardiomyocytes face cytokine-induced mitochondrial dysfunction. This dysfunction, in turn, disrupts multiple mitochondrial energy metabolism pathways, resulting in compromised cell metabolism and myocardium malfunction; additionally, the release of mitochondrial DAMPs could lead to a self-perpetuating positive feedback loop of mitochondrial damage and inflammation. The repercussions of this impairment include diminished ATP production, heightened generation of ROS and RNS, suppression of FA beta-oxidation, and the stimulation of glycolysis activation within cardiomyocytes. These intricate processes collectively impact energy production and play a crucial role in the progression toward CCC.

## 4. Drug Repurposing, New Drug Regimens, and Drug Combinations in Chagas Disease Targeting the Parasite and Pathophysiology of CCC

Following the acute phase, the CCC progresses as a silent disease due to its highly complex pathobiology, where low-grade chronic *T. cruzi* parasitism induces immunopathology, leading to the symptomatic cardiac and digestive forms of the disease in a proportion of infected patients. Genetics, immunology, and metabolic/mitochondrial factors are strictly interlinked to the pathogenesis of this disease; therefore, the identification of compounds that can mitigate the progression of the disease is urgent.

Chagas disease is one of the most neglected diseases and faces restrained funding resources, which negatively impacts research focusing on the identification of new therapeutic compounds beyond the partially effective Benznidazole and Nifurtimox that were licensed over 60 years ago. These drugs present significant adverse effects that compromise adherence to the drug regimen and have not been found to stop clinical progression to severe heart disease in a randomized, controlled clinical trial [7]. CCC has a worse prognosis and is less responsive to heart failure treatment than cardiomyopathies of noninflammatory etiology, like idiopathic or ICs [5]. Novel benznidazole regimens [183], drug repurposing, and drug combination approaches have been tried in recent years to achieve better tolerability and effectiveness.

In comparison to traditional drug discovery, repurposing of drugs already licensed for human use to new clinical indications reduces costs and time of research and development and has ascertained new uses for several compounds for multiple health conditions [184–190]. In the context of Chagas disease, researchers investigated repositioned compounds that could have a standalone or additive effect with benznidazole to increase its anti-*T. cruzi* activity, potentially allowing the administration of lower doses of benznidazole, thus reducing its toxicity and increasing effectiveness [191, 192]. However, several products, including antifungal compounds targeting ergosterol biosynthesis, showed promising anti-*T. cruzi* potential in vitro and in murine models of *T. cruzi* infection, no long-term standalone or additive effect to benznidazole was found on blood parasite DNA levels in clinical trials with antifungals with chronically infected Chagas disease patients [193].

In addition to attempts at improving parasite control or eradication, other investigators investigated therapeutic targets related to the pathophysiology of CCC development in preclinical rodent models of CCC. Anti-cytokine treatment yielded contradictory results; while the use of Infliximab, an anti-TNF-*α* antibody, blocked disease progression in a murine model of CCC [194], TNF-*α* blockade with Etanercept aggravated progression of ventricular dysfunction in a Syrian hamster model of CCC [195], indicating the complexity of anti-inflammatory therapy for Chagas disease, this protective effect of TNF-alpha may have been due to activation-induced cell death of autoreactive CD4+ T cells [196]. On the other hand, targeting downstream elements of cytokine-induced mitochondrial dysfunction may circumvent the potential problems with reducing cytokine activity in a chronic infection that is known to reactivate during immunosuppression. Indeed, several studies in rodent models of CCC achieved protection against chronic cardiac damage by blocking downstream effects of cytokine-induced mitochondrial dysfunction, such as inflammation, nitro-oxidative stress, metabolic/mitochondrial dysfunction, and fibrosis [146, 159, 161, 165, 194]. Wen et al. [197] reported that the nitrone-based antioxidant phenyl-alpha-tert-butylnitrone (PBN) plus benznidazole in murine models of CCC mitigated heart inflammatory and oxidative pathology by decreasing mitochondrial ROS level and oxidative adducts and by preserving the respiratory chain efficiency in Chagasic hearts of rats. Treatment with mitochondria-targeted antioxidant tempol decreased myocardial lipid peroxidation and restored normal heart rhythm and left ventricular dysfunction in murine models of CCC [198]. In vivo treatment with AMPK/NRF2 agonists such as the antidiabetic drug metformin, the flavonoid resveratrol, and SIRT1 agonists targeting mitochondrial FA beta-oxidation, mitochondrial biogenesis, antioxidant response and NF-kB inflammatory signaling led to reduction of cardiac inflammation, oxidative stress and improvement of ventricular function in murine models of CCC [181, 198]. Fenofibrate, the peroxisome proliferator-activated receptor alpha (PPARA) agonist used for dyslipidemia, stimulated mitochondrial FA beta-oxidation, restored left ventricular function, and reduced myocarditis and fibrosis in a murine model of chronic CCC [199]. Treatment with pentoxifylline, a phosphodiesterase inhibitor used for chronic occlusive arterial disease, which has both antioxidant and anti-inflammatory properties, reduced inflammation, and myocardial perfusion defects in a Syrian hamster model of CCC [200]. Cotreatment of mice with benznidazole and pentoxifylline improved electrocardiographic alterations in mice chronically infected with *T. cruzi* [201, 202]. Long-term treatment since early infection with spironolactone, a potassium-sparing diuretic and fibrosis inhibitor [203] and colchicine, an anti-inflammatory drug used for gout [204] significantly reduced chronic phase mortality and heart fibrosis in the Syrian hamster model of CCC. Together, these preclinical studies indicate that several repositioned drugs licensed for human use with known safety profiles and targeting key steps of the pathophysiology of CCC could in theory enter clinical trials for amelioration or blocking progression of Chagas disease cardiomyopathy. Regarding anticytokine treatment, the use of Infliximab, an anti-TNF-*α* antibody, blocked disease progression in a murine model of CCC [205]. On the other hand, TNF-*α* blockade with Etanercept aggravated the progression of ventricular dysfunction in a Syrian hamster model of CCC [195], indicating the complexity of anti-inflammatory therapy for Chagas disease. A recent paper has shown that the protective effect of TNF-*α* in myocarditis could be due to its role in promoting activation-induced cell death of autoreactive CD4+ T cells [196]. Taken together, these preclinical studies indicate that several repositioned drugs licensed for human use with known safety profiles and targeting key steps of the pathophysiology of CCC could, in theory, enter clinical trials for amelioration or blocking the progression of Chagas disease cardiomyopathy. Inasmuch as inflammation, nitro-oxidative stress, and energy metabolism/mitochondrial dysfunction are present and seem to play a major pathogenic role in myocardium from CCC patients [159, 161, 165, 175], the above-mentioned animal model studies should be taken with a grain of salt, since the translation of results from rodent models of CCC to clinical trials in patients has often been frustrating. In addition, the increased prevalence of age-associated cardiovascular risk factors such as arterial hypertension, smoking, or diabetes, which can all contribute to the cardiomyopathy phenotype, is difficult to model in rodents.

In conclusion, Chagas disease remains a significant public health challenge, particularly due to its complex pathogenesis and progression from ASY to symptomatic stages. This manuscript provides an updated overview of the genetic factors and pathogenic mechanisms involved in the development of CCC and the digestive forms of the disease. The review underscores the intricate host–pathogen interactions that drive disease progression, highlighting the role of inflammation and mitochondrial damage. Understanding these mechanisms is crucial for identifying potential therapeutic targets and developing strategies for early intervention, thereby contributing to improved clinical outcomes for patients suffering from Chagas disease. We hope that the insights presented in this review will serve as a foundation for future research and contribute to the broader understanding of Chagas disease within the scientific community.

## Figures and Tables

**Table 1:** Listing of case-controlstudies on association of common gene polymorphisms with susceptibility to different forms of Chagas disease, 1998–2024.

Name	Gene	Gene Classification	Polymorphism	Association	Reference	COHORT / Population
Beta-myosin heavy chain	*MYH7*	Cardiovascular gene	(CATT)n	—	[52]	Brazilian
Alpha-cardiac actin	*ACTC1*	Cardiovascular gene	rs640249	CCC	[50]	Brazilian
Alpha-cardiac actin	*ACTC1*	Cardiovascular gene	rs639735, rs893130, rs475786, rs893131, rs893132, rs533225, rs670957, rs525720, rs7166484, rs2070664; rs3729755, rs533021, rs1851317, rs492038, rs4924215, rs4924214 rs7179902	—	[50]	Brazilian
Voltage-gated sodium channel alpha subunit 5	*SCN5A*	Cardiovascular gene	rs1805124	CCC	[53]	Argentinian
Voltage-gated sodium channel alpha subunit 5	*SCN5A*	Cardiovascular gene	rs36210423	—	[53]	Argentinian
Angiotensin converting enzyme	*ACE*	Cardiovascular gene	I/D	—	[54]	Brazilian
Angiotensin converting enzyme	*ACE*	Cardiovascular gene	I/D	—	[55]	Brazilian
Angiotensin converting enzyme	*ACE*	Cardiovascular gene	DD genotype/D	CCC severity	[56]	Brazilian
CC-chemokine ligand 2	*CCL2*	Chemokine	−2518 (rs1024611)	CCC	[57]	Brazilian
CC-chemokine ligand 2	*CCL2*	Chemokine	rs2530797 *⁣*^*∗∗*^ LD rs10124611	CCC	[49]	Brazilian
CC-chemokine ligand 5	*CCL5*	Chemokine	CCL5 −403 (rs2107538)	CCC	[58]	Brazilian
CC-chemokine ligand 5	*CCL5*	Chemokine	rs3181077, rs1491961, rs3136672, rs2280788	—	[58]	Brazilian
C-X-C motif chemokine ligand 8	*CXCL8*	Chemokine	−251A/T (rs4073)	—	[59]	Colombian
C-X-C motif chemokine ligand 9	*CXCL9*	Chemokine	rs10336	CCC severity	[29]	Brazilian
C-X-C motif chemokine ligand 10	*CXCL10*	Chemokine	rs3921	CCC severity	[29]	Brazilian
CC-chemokine ligand 2	*CCL2*	Chemokine	−2518 (rs1024611)	—	[58]	Brazilian
CC-chemokine ligand 2	*CCL2*	Chemokine	rs3760396, rs2857656, rs4586, rs3917891, rs991804	—	[49]	Brazilian
C–C motif chemokine receptor 1	*CCR1*	Chemokine receptor	rs3181077, rs1491961, rs3136672	—	[58]	Brazilian
C–C motif chemokine receptor 2	*CCR2*	Chemokine receptor	rs1799864	—	[59]	Colombian
C–C motif chemokine receptor 2	*CCR2*	Chemokine receptor	rs1799864	CCC severity	[60]	Colombian
C–C motif chemokine receptor 2	*CCR2*	Chemokine receptor	rs3138042	—	[60]	Colombian
C–C motif chemokine receptor 5	*CCR5*	Chemokine receptor	*Δ*32 rs333	—	[61]	Peruvian
C–C motif chemokine receptor 5	*CCR5*	Chemokine receptor	*Δ*32 rs333	—	[62]	Venezuelan
C–C motif chemokine receptor 5	*CCR5*	Chemokine receptor	*Δ*32 rs333	—	[63]	Brazilian
C–C motif chemokine receptor 5	*CCR5*	Chemokine receptor	CCR5 59029-AG genotype (rs1799987)	Digestive form	[63]	Brazilian
C–C motif chemokine receptor 5	*CCR5*	Chemokine receptor	rs1799987	—	[58]	Brazilian
C–C motif chemokine receptor 5	*CCR5*	Chemokine receptor	rs2856758, rs2734648, rs1799987, rs1799988, rs41469351, rs1800024	—	[60]	Colombian
C–C motif chemokine receptor 5	*CCR5*	Chemokine receptor	rs1799988	CCC severity	[29]	Brazilian
C–C motif chemokine receptor 5	*CCR5*	Chemokine receptor	−2733G	CCC	[59]	Colombian
C–C motif chemokine receptor 5	*CCR5*	Chemokine receptor	−2554 T	CCC severity	[59]	Colombian
C–C motif chemokine receptor 5	*CCR5*	Chemokine receptor	−2459 A/G (rs1799987), −2135 T/C (rs1799988), −2132 C/T (rs41469351), −2086 A/G (rs1800023), −1835 C/T (rs1800024), CCR5−*Δ*32 (rs333)	—	[59]	Colombian
C–C motif chemokine receptor 5-C–C motif chemokine receptor 2	*CCR5-CCR2*	Chemokine receptor	human haplogroup (HH)-A	CCC	[59]	Colombian
C–C motif chemokine receptor 5	*CCR5*	Chemokine receptor	rs1800024	CCC severity	[60]	Colombian
C–C motif chemokine receptor 5	*CCR5*	Chemokine receptor	rs3087253, rs11575815	—	[49]	Brazilian
C–C motif chemokine receptor 5	*CCR5*	Chemokine receptor	CCR5 59029-G (rs1799987)	CCC	[61]	Peruvian
C–C motif chemokine receptor 5	*CCR5*	Chemokine receptor	CCR5 59029-G (rs1799987)	—	[62]	Venezuelan
C–C motif chemokine receptor 5	*CCR5*	Chemokine receptor	rs3176763	CCC	[49]	Brazilian
Interleukin 27B/Epstein-Barr virus induced 3	*EBI3/IL27B*	Cytokine	rs4740, rs4905 genotypes	CCC severity	[31]	Brazilian
Lymphotoxin alpha	*LTA*	Cytokine	+80; +252	CCC	[64]	Brazilian
Lymphotoxin alpha	*LTA*	Cytokine	252	CCC	[65]	Brazilian
Lymphotoxin alpha	*LTA*	Cytokine	rs909253, rs2239704	—	[66]	Brazilian
Transforming growth factor beta 1	*TGFB1*	Cytokine	rs8179181, rs8105161, rs1800469	—	[66]	Brazilian
Transforming growth factor beta 1	*TGFB1*	Cytokine	TGFB1-800, −509, +25, +263	—	[67]	Brazilian
Transforming growth factor beta 1	*TGFB1*	Cytokine	TGFB1 +10	Infection	[68]	Peruvian, Colombian
Transforming growth factor beta 1	*TGFB1*	Cytokine	*TGFB1*-509, *TGFB1*+10	infection	[67]	Brazilian
Interferon gamma	*IFNG*	Cytokine	rs2430561/IFNG +874T/A	Infection	[69]	Colombian
Interferon gamma	*IFNG*	Cytokine	rs2430561	—	[66]	Brazilian
Interleukin 1 alpha	*IL1A*	Cytokine	889C/T; +4845G/T	—	[70]	Colombian
Interleukin 1 beta	*IL1B*	Cytokine	+5810G; +5810GG genotype	CCC	[70]	Colombian
Interleukin 1 beta	*IL1B*	Cytokine	511C/T; 31T/C; +3954T/C	—	[70]	Colombian
interleukin 1 beta	*IL1B*	Cytokine	IL-1B-511, IL-1F10.3	—	[71]	Mexican
Interleukin 4	*IL4*	Cytokine	−590T (rs2243250)	Infection	[72]	Bolivian
Interleukin 4	*IL4*	Cytokine	−590 (rs2243250)	—	[73]	Colombian
Interleukin 6	*IL6*	Cytokine	−174	—	[74]	Colombian
Interleukin 10	*IL10*	Cytokine	−1082	CCC	[75]	Brazilian
Interleukin 10	*IL10*	Cytokine	−1082, −819, −592	—	[73]	Colombian
Interleukin 10	*IL10*	Cytokine	−819 rs1800871TT −592 rs1800872AA genotypes	CCC	[76]	Argentinian
Interleukin 10	*IL10*	Cytokine	−819C>T (rs1800871)	CCC	[76]	Metaanalysis
Interleukin 10	*IL10*	Cytokine	rs1800890, rs1800871, rs1800896	—	[66]	Brazilian
Interleukin 10	*IL10*	Cytokine	rs3024496C	CCC	[51]	Brazilian
Interleukin 12 beta	*IL12B*	Cytokine	1188	CCC	[77]	Colombian
Interleukin 12 beta	*IL12B*	Cytokine	rs2546893, rs919766	CCC	[51]	Brazilian
Interleukin17 alpha	*IL17A*	Cytokine	rs4711998, rs3819024, rs2275913 and rs7747909	—	[78]	Colombian
Interleukin17 alpha	*IL17A*	Cytokine	rs8193036	CCC and infection	[78]	Colombian
Interleukin17 alpha	*IL17A*	Cytokine	rs763780	Infection	[79]	Brazilian
Interleukin 17 alpha	*IL17A*	Cytokine	rs4711998	Infection	[80]	Meta-analysis-Colombian, Bolivian, Argentinian, Brazilian
Interleukin17F	*IL17F*	Cytokine	rs2275913	CCC severity	[79]	Brazilian
Interleukin18	*IL18*	Cytokine	rs2043055, rs1946518 and rs360719	Infection	[81]	Colombian
Interleukin18	*IL18*	Cytokine	rs2043055	CCC	[81]	Colombian
Interleukin18	*IL18*	Cytokine	rs2043055, rs1946518	—	[81]	Meta-analysis-Colombian, Bolivian, Argentinian, Brazilian
Interleukin18	*IL18*	Cytokine	rs360719	Infection	[81]	Meta-analysis-Colombian, Argentinian
Interleukin18	*IL18*	Cytokine	rs5744258, rs360722	—	[82]	Colombian
Interleukin18	*IL18*	Cytokine	rs2043055	CCC severity	[83]	Brazilian
Interleukin18	*IL18*	Cytokine	rs2043055, rs187238, rs1946518 and rs360719	Infection	[82]	Colombian
Macrophage migration inhibitory factor	*MIF*	Cytokine	−173	Infection	[84]	Colombian/Peruvian
Transforming growth factor beta 1	*TGFB1*	Cytokine	+10	Infection	[68]	Peruvian
Transforming growth factor beta 1	*TGFB1*	Cytokine	−509 C>T rs1800469, +10 T>C rs1800470	Infection	[67]	Brazilian
Transforming growth factor beta 1	*TGFB1*	Cytokine	−800 G>A rs1800468,+25 G>C rs1800471, and +263 C>T rs1800472	—	[67]	Brazilian
Transforming growth factor beta 1	*TGFB1*	Cytokine	−988 C/A; −800 G/A; −509 C/T; and 263 C/T	—	[68]	Peruvian, Colombian
TNF alpha	*TNFA*	Cytokine	rs1800629	—	[66]	Brazilian
TNF alpha	*TNFA*	Cytokine	−308	CCC	[85]	Mexican
TNF alpha	*TNFA*	Cytokine	−308	CCC	[4]	Meta-analysis Brazilian, Mexican, Peruvian
TNF alpha	*TNFA*	Cytokine	−308 and TNFa microsatellite	Death	[86]	Brazilian
TNF alpha	*TNFA*	Cytokine	−308 and TNFa microsatellite	—	[87]	Brazilian
TNF alpha	*TNFA*	Cytokine	−238	CCC	[88]	Brazilian
TNF alpha	*TNFA*	Cytokine	−308, −244 and −238	—	[89]	Peruvian
TNF alpha	*TNFA*	Cytokine	−308	—	[88]	Brazilian
TNF alpha	*TNFA*	Cytokine	−546	—	[90]	Peruvian
TNF alpha	*TNFA*	Cytokine	−238, −308, −857, −863, −1031	—	[91]	Bolivian
TNF alpha	*TNFA*	Cytokine	676	—	[92]	Colombian
TNF alpha	*TNFA*	Cytokine	rs1799964, rs1800629	CCC	[92]	Colombian
IL-1 receptor antagonist	*IL1RN*	Cytokine	IL1RN.4	Infection	[71]	Mexican
IL-1 receptor antagonist	*IL1RN*	Cytokine	IL-1RN 6/1, and IL-1RN 6/2	—	[71]	Mexican
IL-1 receptor antagonist	*IL1RN*	Cytokine	IL-1RN (+8006T/C; +8061C/ T; +11100T/C)	—	[70]	Colombian
Interleukin 4 receptor alpha	*IL4RA*	Cytokine receptor	+148 AA genotype	CCC	[73]	Colombian
Tumor necrosis factor Receptor superfamily, member 1	*TNFR1/TNFRSF1A*	Cytokine receptor	rs767455	—	[66]	Brazilian
Tumor necrosis factor receptor superfamily, member 2	*TNFR2/TNFRSF1B*	Cytokine receptor	rs1061624	—	[66]	Brazilian
ADAM metallopeptidase domain 12	*ADAM12*	Extracellular matrix remodeling gene	rs11244787 and rs1871054	Congenital infection	[93]	Argentinian
Matrix metalloproteinase 2	*MMP2*	Extracellular matrix remodeling gene	rs243866, rs17859821, and rs2285053	Congenital infection	[93]	Argentinian
Matrix metallopeptidase 2	*MMP2*	Extracellular matrix remodeling gene	rs243864	—	[93]	Argentinian
Matrix metallopeptidase 9	*MMP9*	Extracellular matrix remodeling gene	rs3918242, rs2234681	—	[93]	Argentinian
major histocompatibility complex, class I and II	*HLA*	HLA	Several, class I and II	Contradictory	[52, 91, 94–100]	Brazilian/ Peruvian/venezuelan/Mexican/Bolivian
Mitochondrial ribosomal small subunit18B	*MRPSB18*	Mitochondrial gene	rs34315095	Digestive form (Megaesophagus)	[101]	Brazilian
Vasoactive intestinal peptide receptor 1	*VIPR1*	Other	rs342511T	CCC	[102]	Brazilian
Vasoactive intestinal peptide receptor 2	*VIPR2*	Other	rs885861	CCC	[102]	Brazilian
Haptoglobin	*HP*	Other	HP2	CCC severity	[103]	Brazilian
Haptoglobin	*HP*	Other	HP	CCC	[104]	Venezuelan
SAC3 domain containing 1	*SAC3D1*	Other	rs2458298	CCC	[105]	Colombian, Argentinian, Bolivian and Brazilian (GWAS)
Caspase1	*CASP1*	Other innate immunity genes	rs104936010, rs12417050	—	[106]	Bolivian
Caspase activation recruitment domain 11	*CARD11*	Other innate immunity genes	rs6953573, rs11982651, rs1621828, rs6461749	—	[106]	Bolivian
Collectin-11	*COLEC11*	Other innate immunity genes	rs7567833G//genotypes rs7567833AG and rs7567833GG//COLEC11*⁣*^*∗*^GGC haplotype	Infection, CCC, cardiodigestive form	[107]	Brazilian
Complement receptor type 1	*CR1*	Other innate immunity genes	rs17047660, rs17047661, rs6691117, CR1*⁣*^*∗*^AGAGTG haplotype	Infection	[108]	Brazilian
Complement receptor type 1	*CR1*	Other innate immunity genes	CR1*⁣*^*∗*^AGGGTG haplotype	CCC	[108]	Brazilian
Complement receptor type 1	*CR1*	Other innate immunity genes	rs17259045, rs41274768, rs4844609	—	[108]	Brazilian
Cytotoxic T-lymphocyte associated protein 4	*CTLA4*	Other innate immunity genes	rs733618	CCC	[109]	Brazilian
Cytotoxic T-lymphocyte associated protein 4	*CTLA4*	Other innate immunity genes	rs5742909	Cardiodigestive form	[109]	Brazilian
Cytotoxic T-lymphocyte associated protein 4	*CTLA4*	Other innate immunity genes	rs231775	—	[109]	Brazilian
Cytotoxic T-lymphocyte associated protein 4	*CTLA4*	Other innate immunity genes	rs231775	—	[110]	enezuelan
Ficolin-2	*FCN2*	Other innate immunity genes	rs17514136	CCC	[111]	Brazilian
Ficolin-2	*FCN2*	Other innate immunity genes	rs3124952, rs3124953, rs17514136	—	[111]	Brazilian
Ficolin-3	*FCN3*	Other innate immunity genes	rs532781899, rs28362807 and rs4494157	—	[112]	Brazilian
Forkhead box O3	*FOXO3*	Other innate immunity genes	rs12212067	—	[113]	Colombian
Galectin 3	*LGALS3*	Other innate immunity genes	rs4644 and rs4652	—	[114]	Brazilian
Inducible nitric oxide synthase	*NOS2*	Other innate immunity genes	(CCTTT)n	—	[90]	Peruvian
Killer cell immunoglobulin-like receptors	*KIR*	Other innate immunity genes	KIR2DS2−//KIR2DL2−/Haplotype KIR2DL3+/C1	Digestive form	[115]	Brazilian
Mannose binding lectin 2	*MBL2*	Other innate immunity genes	MBL2*⁣*^*∗*^B	CCC	[116]	Chilean
MBL-associated serine protease 2	*MASP2*	Other innate immunity genes	g.1961795C p.371D diplotype, MASP2*⁣*^*∗*^CD genotypes, g.1945560A	CCC	[117]	Brazilian
MBL-associated serine protease 2	*MASP2*	Other innate immunity genes	rs1961795	CCC and digestive form	[107]	Brazilian
megakaryoblastic leukemia/MyD88 adaptor-like protein	*TIRAP*	Other innate immunity genes	S180L (rs8177374)	CCC	[118]	Brazilian
megakaryoblastic leukemia/MyD88 adaptor-like protein	*TIRAP*	Other innate immunity genes	S180L (rs8177374)	—	[116]	Chilean
megakaryoblastic leukemia/MyD88 adaptor-like protein	*TIRAP*	Other innate immunity genes	rs8177376A/A*⁣*^*∗∗*^ LD rs8177374	CCC	[49]	Brazilian
megakaryoblastic leukemia/MyD88 adaptor-like protein	*TIRAP*	Other innate immunity genes	rs11220437, rs591163, rs8177352, rs8177375, rs17866704	—	[49]	Brazilian
Killer cell immunoglobulin-like receptors	*KIR*	Other innate immunity genes	*KIR2DS2+/KIRD2L2-/HLA-C1*	CCC, CCC severity	[119]	Brazilian
MHC class I polypeptide-related sequence A	*MICA*	Other innate immunity genes	MICA-129 Met/Met (rs1051792)	CCC severity	[120]	Brazilian
MHC class I-related chain A	*MICA*	Other innate immunity genes	MICA-129 Met (rs1051792)	CCC Severity	[115]	Brazilian
MHC class I-related chain A	*MICA*	Other innate immunity genes	haplotype MICA*⁣*^*∗*^008~HLA-C*⁣*^*∗*^06	Digestive form	[115]	Brazilian
Natural resistance associated macrophage protein 1	*NRAMP1*	Other innate immunity genes	5'(GT)n, −236 C–>T, D543N, 3'UTR deletion	—	[121]	Peruvian
NF-*k*B inhibitor like 1	*NFkBIL1*	Other innate immunity genes	−324	CCC	[122]	Brazilian
Nucleotide-binding oligomerization domain-like	*NLRP1*	Other innate immunity genes	rs11651270 missense mutation	CCC	[106]	Bolivian
Nucleotide-binding oligomerization domain-like	*NLRP1*	Other innate immunity genes	rs9303193, rs2301582.	—	[106]	Bolivian
Nucleotide-binding oligomerization domain-like	*NLRP1*	Other innate immunity genes	rs11651270, rs9303193, rs2301582	—	[106]	Bolivian
Programmed cell death 1	*PDCD1*	Other innate immunity genes	rs11568821	—	[109]	Brazilian
Protein tyrosine phosphatase, non receptor type 22	*PTPN22*	Other innate immunity genes	rs1858	—	[123]	Colombian and Peruvian
Toll like receptor 1	*TLR1*	Other innate immunity genes	602	—	[118]	Brazilian
Toll like receptor 1	*TLR1*	Other innate immunity genes	602	—	[116]	Chile
Toll like receptor 2	*TLR2*	Other innate immunity genes	753	—	[124]	Colombian
Toll like receptor 2	*TLR2*	Other innate immunity genes	753	—	[118]	Brazilian
Toll like receptor 2	*TLR2*	Other innate immunity genes	753	—	[116]	Chile
Toll like receptor 4	*TLR4*	Other innate immunity genes	D299G/T399I genotype	CCC	[116]	Chilean
Toll like receptor 4	*TLR4*	Other innate immunity genes	229	—	[118]	Brazilian
Toll like receptor 4	*TLR4*	Other innate immunity genes	229	—	[124]	Colombian
Toll like receptor 4	*TLR4*	Other innate immunity genes	D299G/T399I genotype or 299/399 haplotype	Infection	[125]	Venezuelan
Toll like receptor 4	*TLR4*	Other innate immunity genes	D299G/T399I genotype	CCC	[125]	Venezuelan
Toll like receptor 5	*TLR5*	Other innate immunity genes	392	—	[118]	Brazilian
Toll like receptor 6	*TLR6*	Other innate immunity genes	−249	—	[116]	Chile
Toll like receptor 9	*TLR9*	Other innate immunity genes	−1237 and −1486	—	[118]	Brazilian
Tyrosine kinase 2	*TYK2*	Other innate immunity genes	rs34536443, rs1272035, rs2304256	—	[126]	Colombian
Tyrosine kinase 2	*TYK2*	Other innate immunity genes	rs34536443, rs12720356 and rs2304256	—	[126]	Colombian
phosphatidylinositol-4,5-bisphosphate 3-kinase gamma	*PIK3CG*	Other innate immunity genes	rs1129293	CCC	[127]	Brazilian
Caspase 1	*CASP1*	Other innate immunity genes	rs501192	CCC severity (trend)	[128]	Bolivian
Complement 2 factor	*C2*	Other innate immunity genes	C2	—	[129]	Brazilian
Complement 3 factor	*C3*	Other innate immunity genes	C3F/C3F	CCC	[129]	Brazilian
Complement 4 factor	*C4*	Other innate immunity genes	C4A, C4B	—	[129]	Brazilian
Complement BF factor	*BF*	Other innate immunity genes	BFS	Infection	[129]	Brazilian
DExD-Box Helicase 39B	*DDX39B/BAT1*	Other innate immunity genes	−22, −348	CCC	[130]	Brazilian
DExD-Box Helicase 39B	*DDX39B/BAT1*	Other innate immunity genes	rs3853601	—	[66]	Brazilian

*Note*: Association: Infection, when polymorphism was significant when comparing Chagas disease vs noninfected controls; CCC, when polymorphism was significant when comparing CCC vs IF/ASY; severe CCC (ventricular dysfunction; left ventricular ejection fraction 0.40) vs. severe CCC (comparisons of severe CCC versus ASY were not computed); digestive/cardiodigestive, when polymorphism was significant when comparing digestive (megacolon and/or megaesophagus; diagnosed with X-ray analysis by barium enema and barium esophagography and manometry)/cardiodigestive disease with IF/ASY. For the sake of simplicity, all the polymorphisms cited in the table are described as risk factors for disease or disease progression (i. e., an odds ratio >1), even when the publication emphasizes the protective allele/genotype (an odds ratio below 1). This can be done due to the complementarity of base pairs.

**Table 2:** IFN-*γ* and TNF-*α* treatment of AC16 cardiomyocytes recapitulates findings in CCC heart tissue.

Cytokines/pathways	Cytokine-treated AC16 cardiomyocytes	CCC myocardium findings
*Cytokine effects*		
- Mitochondrial membrane potential	**↓**	—
- ATP/high-energy phosphates	**↓**	**↓**
- Fatty acid *β*-oxidation	**↓**	**↓**
- mtDNA copy number	**↓**	**↓**
- Expression of TCA, OXPHOS, fatty acid *β*-oxidation, and - creatine kinase enzymes	**↓**	**↓**
- Nitro-oxidative stress	**↑**	**↑**
*Pathways analysis*		
- IFN-*γ* signaling	**↑**	**↑**
- Mitochondrial dysfunction	**↑**	**↑**
-Transmembrane potential	**↓**	**↓**
- Respiratory electron transport/OXPHOS	**↓**	**↓**
Citric acid cycle (TCA cycle)	**↓**	**↓**
- Fatty acid *β*-oxidation	**↓↓**	**↓↓**
- Glycolysis	**↑**	**↑**
- Mitochondrial protein import	**↓**	**↓**

*Note:* Cytokine effects refer to cytokine-induced phenotypes on AC16 cardiomyocytes and whether these phenotypes also occurred in CCC myocardium. Cytokine effects and pathways analysis performed on proteomic and transcriptomic data from CCC myocardium and cytokine-treated AC16 cardiomyocytes come from references [28, 161, 165].

## Data Availability

The data supporting this review are available from the corresponding author upon reasonable request.

## References

[1] Pérez-Molina J. A., Molina I. (2018). Chagas Disease. *The Lancet*.

[2] Chagas disease - PAHO/WHO | Pan American Health Organization (2024). https://www.paho.org/en/topics/chagas-disease.

[3] Maladie de Chagas (ou trypanosomiase américaine) https://www.who.int/fr/news-room/fact-sheets/detail/chagas-disease-(american-trypanosomiasis).

[4] Cunha-Neto E., Chevillard C. (2014). Chagas Disease Cardiomyopathy: Immunopathology and Genetics. *Mediators of Inflammation*.

[5] Bestetti R. B., Muccillo G. (1997). Clinical Course of Chagas’ Heart Disease: A Comparison With Dilated Cardiomyopathy. *International Journal of Cardiology*.

[6] Pérez-Molina J. A., Crespillo-Andújar C., Bosch-Nicolau P., Molina I. (2021). Trypanocidal Treatment of Chagas Disease. *Enfermedades Infecciosas y Microbiología Clínica*.

[7] Morillo C. A., Marin-Neto J. A., Avezum A. (2015). Randomized Trial of Benznidazole for Chronic Chagas’ Cardiomyopathy. *New England Journal of Medicine*.

[8] De Higuchi M. L., De Floriano Morais C., Barreto A. C. P. (1987). The Role of Active Myocarditis in the Development of Heart Failure in Chronic Chagas’ Disease: A Study Based on Endomyocardial Biopsies. *Clinical Cardiology*.

[9] da Matta Guedes P. M., Gutierrez F. R. S., Maia F. L. (2010). IL-17 Produced During *Trypanosoma cruzi* Infection Plays a Central Role in Regulating Parasite-Induced Myocarditis. *PLoS Neglected Tropical Diseases*.

[10] Rodrigues M. M., Oliveira A. C., Bellio M. (2012). The Immune Response to *Trypanosoma cruzi*: Role of Toll-Like Receptors and Perspectives for Vaccine Development. *Journal of Parasitology Research*.

[11] Abel L. C., Rizzo L. V., Ianni B. (2001). Chronic Chagas’ Disease Cardiomyopathy Patients Display an Increased IFN-Gamma Response to *Trypanosoma cruzi* Infection. *Journal of Autoimmunity*.

[12] Gomes J. A. S., Bahia-Oliveira L. M. G., Rocha M. O. C., Martins-Filho O. A., Gazzinelli G., Correa-Oliveira R. (2003). Evidence that Development of Severe Cardiomyopathy in Human Chagas’ Disease Is Due to a Th1-Specific Immune Response. *Infection and Immunity*.

[13] Chevillard C., Nunes J. P. S., Frade A. F. (2018). Disease Tolerance and Pathogen Resistance Genes May Underlie *Trypanosoma cruzi* Persistence and Differential Progression to Chagas Disease Cardiomyopathy. *Frontiers in Immunology*.

[14] Gomes J. A. S., Bahia-Oliveira L. M. G., Rocha M. O. C. (2005). Type 1 Chemokine Receptor Expression in Chagas’ Disease Correlates With Morbidity in Cardiac Patients. *Infection and Immunity*.

[15] Sousa G. R., Gomes J. A. S., Fares R. C. G. (2014). Plasma Cytokine Expression Is Associated With Cardiac Morbidity in Chagas Disease. *PLoS ONE*.

[16] Ferreira R. C., Ianni B. M., Abel L. C. J. (2003). Increased Plasma Levels of Tumor Necrosis Factor-Alpha in Asymptomatic/“indeterminate” and Chagas Disease Cardiomyopathy Patients. *Memórias do Instituto Oswaldo Cruz*.

[17] Cunha-Neto E., Teixeira P. C., Nogueira L. G., Kalil J. (2011). Autoimmunity. *Advances in Parasitology*.

[18] Rizzo L. V., Cunha-Neto E., Teixeira A. R. (1989). Autoimmunity in Chagas’ Disease: Specific Inhibition of Reactivity of CD4+ T Cells Against Myosin in Mice Chronically Infected With Trypanosoma Cruzi. *Infection and Immunity*.

[19] Tibbetts R. S., McCormick T. S., Rowland E. C., Miller S. D., Engman D. M. (1994). Cardiac Antigen-Specific Autoantibody Production Is Associated With Cardiomyopathy in Trypanosoma Cruzi-Infected Mice. *The Journal of Immunology*.

[20] Leon J. S., Daniels M. D., Toriello K. M., Wang K., Engman D. M. (2004). A Cardiac Myosin-Specific Autoimmune Response Is Induced by Immunization With *Trypanosoma cruzi* Proteins. *Infection and Immunity*.

[21] Abel L. C. J., Kalil J., Cunha-Neto E. (1997). Molecular Mimicry Between Cardiac Myosin and Trypanosoma Cruzi Antigen B13: Identification of a B13-Driven Human T Cell Clone That Recognizes Cardiac Myosin. *Brazilian Journal of Medical and Biological Research*.

[22] Abel L. C. J., Iwai L. K., Viviani W. (2005). T Cell Epitope Characterization in Tandemly Repetitive *Trypanosoma cruzi* B13 Protein. *Microbes and Infection*.

[23] Iwai L. K., Juliano M. A., Juliano L., Kalil J., Cunha-Neto E. T-Cell Molecular Mimicry in Chagas Disease: Identification and Partial Structural Analysis of Multiple Cross-Reactive Epitopes Between *Trypanosoma cruzi* B13 and Cardiac Myosin Heavy Chain. *Journal of Autoimmunity*.

[24] Reis M. M., de Lourdes Higuchi M., Benvenuti L. A. (1997). An in Situ Quantitative Immunohistochemical Study of Cytokines and IL-2R^+^ in Chronic Human Chagasic Myocarditis: Correlation With the Presence of Myocardial *Trypanosoma cruzi* Antigens. *Clinical Immunology and Immunopathology*.

[25] Rodrigues D. B. R., dos Reis M. A., Romano A. (2012). *In Situ* Expression of Regulatory Cytokines by Heart Inflammatory Cells in Chagas’ Disease Patients With Heart Failure. *Clinical and Developmental Immunology*.

[26] Nogueira L. G., Santos R. H. B., Fiorelli A. I. (2014). Myocardial Gene Expression of *T-bet*, *GATA-3*, *Ror- γ* t, *FoxP3*, and Hallmark Cytokines in Chronic Chagas Disease Cardiomyopathy: An Essentially Unopposed T_H_ 1-Type Response. *Mediators of Inflammation*.

[27] Cunha-Neto E., Dzau V. J., Allen P. D. (2005). Cardiac Gene Expression Profiling Provides Evidence for Cytokinopathy as a Molecular Mechanism in Chagas’ Disease Cardiomyopathy. *The American Journal of Pathology*.

[28] Laugier L., Ferreira L. R. P., Ferreira F. M. (2020). MiRNAs May Play a Major Role in the Control of Gene Expression in Key Pathobiological Processes in Chagas Disease Cardiomyopathy. *PLOS Neglected Tropical Diseases*.

[29] Nogueira L. G., Santos R. H. B., Ianni B. M. (2012). Myocardial Chemokine Expression and Intensity of Myocarditis in Chagas Cardiomyopathy Are Controlled by Polymorphisms in CXCL9 and CXCL10. *PLoS Neglected Tropical Diseases*.

[30] Torzewski M., Wenzel P., Kleinert H. (2012). Chronic Inflammatory Cardiomyopathy of Interferon *γ*-Overexpressing Transgenic Mice Is Mediated by Tumor Necrosis Factor-*α*. *The American Journal of Pathology*.

[31] Medina T. S., Oliveira G. G., Silva M. C. (2017). Ebi3 Prevents Trypanosoma Cruzi-Induced Myocarditis by Dampening IFN-*γ*-Driven Inflammation. *Frontiers in Immunology*.

[32] Sunderraj A., Cunha L. M., Avila M. (2024). Parasite DNA and Markers of Decreased Immune Activation Associate Prospectively With Cardiac Functional Decline Over 10 Years Among *Trypanosoma cruzi* Seropositive Individuals in Brazil. *International Journal of Molecular Sciences*.

[33] Ferreira L. R. P., Ferreira F. M., Nakaya H. I. (2017). Blood Gene Signatures of Chagas Cardiomyopathy With or Without Ventricular Dysfunction. *The Journal of Infectious Diseases*.

[34] Sabino E. C., Ribeiro A. L., Lee T. H. (2015). Detection of *Trypanosoma cruzi* DNA in Blood by PCR Is Associated With Chagas Cardiomyopathy and Disease Severity. *European Journal of Heart Failure*.

[35] Cardoso C. S., Ribeiro A. L. P., Oliveira C. D. L. (2018). Beneficial Effects of Benznidazole in Chagas Disease: NIH SaMi-Trop Cohort Study. *PLOS Neglected Tropical Diseases*.

[36] de Oliveira M. T., Schmidt A., da Silva M. C., Donadi E. A., da Silva J. S., Marin-Neto J. A. (2021). Parasitic Load Correlates With Left Ventricular Dysfunction in Patients With Chronic Chagas Cardiomyopathy. *Frontiers in Cardiovascular Medicine*.

[37] Cutrullis R. A., Moscatelli G. F., Moroni S. (2011). Benzonidazole Therapy Modulates Interferon-*γ* and M2 Muscarinic Receptor Autoantibody Responses in Trypanosoma Cruzi-Infected Children. *PLoS ONE*.

[38] Laucella S. A., Mazliah D. P., Bertocchi G. (2009). Changes in *Trypanosoma cruzi* –Specific Immune Responses after Treatment: Surrogate Markers of Treatment Efficacy. *Clinical Infectious Diseases*.

[39] Silva J. S., Vespa G. N., Cardoso M. A., Aliberti J. C., Cunha F. Q. (1995). Tumor Necrosis Factor Alpha Mediates Resistance to Trypanosoma Cruzi Infection in Mice by Inducing Nitric Oxide Production in Infected Gamma Interferon-Activated Macrophages. *Infection and Immunity*.

[40] Ferreira L. R. P., Frade A. F., Baron M. A. (2014). Interferon-*γ* and Other Inflammatory Mediators in Cardiomyocyte Signaling During Chagas Disease Cardiomyopathy. *World Journal of Cardiology*.

[41] Diehl S., Rincón M. (2002). The Two Faces of IL-6 on Th1/Th2 Differentiation. *Molecular Immunology*.

[42] Viana C. E. M., Matos D. M., de Fátima Oliveira M. (2021). Immunosuppressive CD14+/HLA-DRlow/‒ Monocytes in Patients With Chagas Disease. *Acta Tropica*.

[43] Pérez-Mazliah D. E., Castro Eiro M. D., Álvarez M. G. (2018). Distinct Monocyte Subset Phenotypes in Patients With Different Clinical Forms of Chronic Chagas Disease and Seronegative Dilated Cardiomyopathy. *PLoS Neglected Tropical Diseases*.

[44] Ribeiro B. M., Crema E., Rodrigues V. (2008). Analysis of the Cellular Immune Response in Patients With the Digestive and Indeterminate Forms of Chagas’ Disease. *Human Immunology*.

[45] de Sena Pereira N., Queiroga T. B. D., Nunes D. F. (2018). Innate Immune Receptors Over Expression Correlate With Chronic Chagasic Cardiomyopathy and Digestive Damage in Patients. *PLOS Neglected Tropical Diseases*.

[46] da Silveira A. B. M., Arantes R. M. E., Vago A. R. (2005). Comparative Study of the Presence of Trypanosoma Cruzi kDNA, Inflammation and Denervation in Chagasic Patients With and Without Megaesophagus. *Parasitology*.

[47] Ribeiro B. M., Helmo F. R., Rodrigues D. B. R., Silva M. V. D., Rodrigues V. (2025). Higher T-bet and IFN-*γ* Expression in Advanced Chagasic Megaesophagus Indicates Th1 Response in the Chronic Phase. *Revista do Instituto de Medicina Tropical de São Paulo*.

[48] Acosta-Herrera M., Strauss M., Casares-Marfil Dé, Martín J. (2019). Genomic Medicine in Chagas Disease. *Acta Tropica*.

[49] Frade A. F., Pissetti C. W., Ianni B. M. (2013). Genetic Susceptibility to Chagas Disease Cardiomyopathy: Involvement of Several Genes of the Innate Immunity and Chemokine-Dependent Migration Pathways. *BMC Infectious Diseases*.

[50] Frade A. F., Teixeira P. C., Ianni B. M. (2013). Polymorphism in the Alpha Cardiac Muscle Actin 1 Gene Is Associated to Susceptibility to Chronic Inflammatory Cardiomyopathy. *PLoS ONE*.

[51] Frade-Barros A. F., Ianni B. M., Cabantous S. (2020). Polymorphisms in Genes Affecting Interferon-*γ* Production and Th1 T Cell Differentiation Are Associated With Progression to Chagas Disease Cardiomyopathy. *Frontiers in Immunology*.

[52] Faé K. C., Drigo S. A., Cunha-Neto E. (2000). HLA and *β*-Myosin Heavy Chain Do Not Influence Susceptibility to Chagas’ Disease Cardiomyopathy. *Microbes and Infection*.

[53] Blasco R. L., Strauss M., Velázquez López D. A. (2021). SCN5A Gene Variants as Potential Markers of the Progression of Chronic Chagasic Cardiac Alterations. *Parasitology International*.

[54] Pascuzzo-Lima C., Mendible J. C., Bonfante-Cabarcas R. A. (2009). Angiotensin-Converting Enzyme Insertion/Deletion Gene Polymorphism and Progression of Chagas’ Cardiomyopathy. *Revista Española de Cardiología*.

[55] da Silva S. J., Rassi S., da Costa Pereira A. (2017). Angiotensin-Converting Enzyme ID Polymorphism in Patients With Heart Failure Secondary to Chagas Disease. *Arquivos Brasileiros de Cardiologia*.

[56] Alves S. M. M., Alvarado-Arnês L. E., da Glória Aureliano de Melo Cavalcanti M. (2020). Influence of Angiotensin-Converting Enzyme Insertion/Deletion Gene Polymorphism in Progression of Chagas Heart Disease. *Revista da Sociedade Brasileira de Medicina Tropical*.

[57] Ramasawmy R., Cunha-Neto E., Fae K. C. (2006). The Monocyte Chemoattractant Protein-1 Gene Polymorphism Is Associated With Cardiomyopathy in Human Chagas Disease. *Clinical Infectious Diseases*.

[58] Batista A. M., Alvarado-Arnez L. E., Alves S. M. (2018). Genetic Polymorphism at CCL5 Is Associated With Protection in Chagas’ Heart Disease: Antagonistic Participation of CCR1+ and CCR5+ Cells in Chronic Chagasic Cardiomyopathy. *Frontiers in Immunology*.

[59] Flórez O., Martín J., González C. I. (2012). Genetic Variants in the Chemokines and Chemokine Receptors in Chagas Disease. *Human Immunology*.

[60] Machuca M. A., Suárez E. U., Echeverría L. E., Martín J., González C. I. (2014). SNP/Haplotype Associations of *CCR2* and *CCR5* Genes With Severity of Chagasic Cardiomyopathy. *Human Immunology*.

[61] Calzada J. E., Nieto A., Beraún Y., Martín J. (2001). Chemokine Receptor CCR5 Polymorphisms and Chagas’ Disease Cardiomyopathy. *Tissue Antigens*.

[62] Fernández-Mestre M. T., Montagnani S., Layrisse Z. (2004). Is the CCR5-59029-G/G Genotype a Protective Factor for Cardiomyopathy in Chagas Disease?. *Human Immunology*.

[63] de Oliveira A. P., Bernardo C. R., Vitória da Silveira Camargo A. (2015). Genetic Susceptibility to Cardiac and Digestive Clinical Forms of Chronic Chagas Disease: Involvement of the CCR5 59029 A/G Polymorphism. *PLoS ONE*.

[64] Ramasawmy R., Fae K. C., Cunha-Neto E. (2007). Polymorphisms in the Gene for Lymphotoxin-*α* Predispose to Chronic Chagas Cardiomyopathy. *The Journal of Infectious Diseases*.

[65] Pissetti C. W., de Oliveira R. F., Correia D., Nascentes G. A. N., Llaguno M. M., Rodrigues V. (2013). Association Between the Lymphotoxin-Alpha Gene Polymorphism and Chagasic Cardiopathy. *Journal of Interferon & Cytokine Research*.

[66] Alvarado-Arnez L. E., Batista A. M., Alves S. M. (2018). Single Nucleotide Polymorphisms of Cytokine-Related Genes and Association With Clinical Outcome in a Chagas Disease Case-Control Study From Brazil. *Memórias do Instituto Oswaldo Cruz*.

[67] Ferreira R. R., da Silva Madeira F., Alves G. F. (2018). TGF-*β* Polymorphisms Are a Risk Factor for Chagas Disease. *Disease Markers*.

[68] Calzada J. E., Beraún Y., González C. I., Martín J. (2009). Transforming Growth Factor Beta 1 (TGF*β*1) Gene Polymorphisms and Chagas Disease Susceptibility in Peruvian and Colombian Patients. *Cytokine*.

[69] Torres O. A., Calzada J. E., Beraún Y. (2010). Role of the IFNG +874T/A Polymorphism in Chagas Disease in a Colombian Population. *Infection, Genetics and Evolution*.

[70] Flórez O., Zafra G., Morillo C., Martín J., González C. I. (2006). Interleukin-1 Gene Cluster Polymorphism in Chagas Disease in a Colombian Case-Control Study. *Human Immunology*.

[71] Cruz-Robles D., Chávez-González J. P., Cavazos-Quero M. M., Pérez-Méndez O., Reyes P. A., Vargas-Alarcón G. (2009). Association Between *IL-1B* and *IL-1RN* Gene Polymorphisms and Chagas’ Disease Development Susceptibility. *Immunological Investigations*.

[72] Alvarado Arnez L. E., Venegas E. N., Ober C., Thompson E. E. (2011). Sequence Variation in the IL4 Gene and Resistance to Trypanosoma Cruzi Infection in Bolivians. *Journal of Allergy and Clinical Immunology*.

[73] Flórez O., Martín J., González C. I. (2011). Interleukin 4, Interleukin 4 Receptor-*α* and Interleukin 10 Gene Polymorphisms in Chagas Disease. *Parasite Immunology*.

[74] Torres O. A., Calzada J. E., Beraún Y. (2010). Lack of Association Between *IL-6*-174G/C Gene Polymorphism and Chagas Disease. *Tissue Antigens*.

[75] Costa G. C., da Costa Rocha M. O., Moreira P. R. (2009). Functional IL-10 Gene Polymorphism Is Associated With Chagas Disease Cardiomyopathy. *The Journal of Infectious Diseases*.

[76] Grijalva A., Gallo Vaulet L., Agüero R. N. (2022). Interleukin 10 Polymorphisms as Risk Factors for Progression to Chagas Disease Cardiomyopathy: A Case-Control Study and Meta-Analysis. *Frontiers in Immunology*.

[77] Zafra G., Morillo C., Martín J., González A., González C. I. (2007). Polymorphism in the 3′ UTR of the *IL12B* Gene Is Associated With Chagas’ Disease Cardiomyopathy. *Microbes and Infection*.

[78] Rodriguez D. A. L., Echeverría L. E., González C. I., Martin J. (2015). Investigation of the Role of IL17A Gene Variants in Chagas Disease. *Genes & Immunity*.

[79] Reis P. G., Ayo C. M., de Mattos L. C. (2017). Genetic Polymorphisms of *IL17* and Chagas Disease in the South and Southeast of Brazil. *Journal of Immunology Research*.

[80] Strauss M., Palma-Vega M., Casares-Marfil D. (2020). Genetic Polymorphisms of IL17A Associated With Chagas Disease: Results From a Meta-Analysis in Latin American Populations. *Scientific Reports*.

[81] Strauss M., Acosta-Herrera M., Alcaraz A. (2019). Association of IL18 Genetic Polymorphisms With Chagas Disease in Latin American Populations. *PLoS Neglected Tropical Diseases*.

[82] Rodriguez D. A. L., Carmona F. D., Echeverría L. E., González C. I., Martin J., Hirayama K. (2016). IL18 Gene Variants Influence the Susceptibility to Chagas Disease. *PLOS Neglected Tropical Diseases*.

[83] Nogueira L. G., Frade A. F., Ianni B. M. (2015). Functional IL18 Polymorphism and Susceptibility to Chronic Chagas Disease. *Cytokine*.

[84] Torres O. A., Calzada J. E., Beraún Y. (2009). Association of the Macrophage Migration Inhibitory Factor -173G/C Polymorphism With Chagas Disease. *Human Immunology*.

[85] Rodríguez-Pérez J. M., Cruz-Robles D., Hernández-Pacheco G. (2005). Tumor Necrosis Factor-Alpha Promoter Polymorphism in Mexican Patients With Chagas’ Disease. *Immunology Letters*.

[86] Drigo S. A., Cunha-Neto E., Ianni B. (2006). TNF Gene Polymorphisms Are Associated With Reduced Survival in Severe Chagas’ Disease Cardiomyopathy Patients. *Microbes and Infection*.

[87] Drigo S. A., Cunha-Neto E., Ianni B. (2007). Lack of Association of Tumor Necrosis Factor-Alpha Polymorphisms With Chagas Disease in Brazilian Patients. *Immunology Letters*.

[88] Pissetti C. W., Correia D., de Oliveira R. F. (2011). Genetic and Functional Role of TNF-Alpha in the Development Trypanosoma Cruzi Infection. *PLoS Neglected Tropical Diseases*.

[89] Beraún Y., Nieto A., Collado M. D., González A., Martín J. (1998). Polymorphisms at Tumor Necrosis Factor (TNF) Loci Are Not Associated With Chagas’ Disease. *Tissue Antigens*.

[90] Calzada J. E., López-Nevot M. A., Beraún Y., Martín J. (2002). No Evidence for Association of the Inducible Nitric Oxide Synthase Promoter Polymorphism With *Trypanosoma cruzi* Infection. *Tissue Antigens*.

[91] del Puerto F., Nishizawa J. E., Kikuchi M. (2012). Protective Human Leucocyte Antigen Haplotype, HLA-DRB1∗01-B^*^14, Against Chronic Chagas Disease in Bolivia. *PLoS Neglected Tropical Diseases*.

[92] Criado L., Flórez O., Martín J., González C. I. (2012). Genetic Polymorphisms in TNFA/TNFR2 Genes and Chagas Disease in a Colombian Endemic Population. *Cytokine*.

[93] Juiz N. A., Cayo N. M., Burgos M. (2016). Human Polymorphisms in Placentally Expressed Genes and Their Association With Susceptibility to Congenital *Trypanosoma cruzi* Infection. *Journal of Infectious Diseases*.

[94] Ayo C. M., de Oliveira Dalalio M. M., Visentainer J. E. L. (2013). Genetic Susceptibility to Chagas Disease: An Overview About the Infection and About the Association Between Disease and the Immune Response Genes. *BioMed Research International*.

[95] Deghaide N. H. S., Dantas R. O., Donadi E. A. (1998). HLA Class I and II Profiles of Patients Presenting With Chagas’ Disease. *Digestive Diseases and Sciences*.

[96] Fernandez-Mestre M. T., Layrisse Z., Montagnani S. (1998). Influence of the HLA Class II Polymorphism in Chronic Chagas’ Disease. *Parasite Immunology*.

[97] Nieto A., Beraún Y., Collado M. D. (2000). HLA Haplotypes Are Associated With Differential Susceptibility to Trypanosoma Cruzi Infection. *Tissue Antigens*.

[98] Layrisse Z., Fernandez M. T., Montagnani S. (2000). HLA-C(∗)03 Is a Risk Factor for Cardiomyopathy in Chagas Disease. *Human Immunology*.

[99] Colorado I. A., Acquatella H., Catalioti F., Fernandez M. T., Layrisse Z. (2000). HLA Class II DRB1, DQB1, DPB1 Polymorphism and Cardiomyopathy due to Trypanosoma Cruzi Chronic Infection. *Human Immunology*.

[100] Cruz-Robles D., Reyes P. A., Monteón-Padilla V. M., Ortiz-Muñiz A. R., Vargas-Alarcón G. (2004). MHC Class I and Class II Genes in Mexican Patients With Chagas Disease. *Human Immunology*.

[101] Silva K. D. A., Nunes J. P. S., Andrieux P. (2022). Chagas Disease Megaesophagus Patients Carrying Variant MRPS18B P260A Display Nitro-Oxidative Stress and Mitochondrial Dysfunction in Response to IFN-*γ* Stimulus. *Biomedicines*.

[102] Corrêa M. V., da Costa Rocha M. O., de Sousa G. R. (2013). Low Levels of Vasoactive Intestinal Peptide Are Associated With Chagas Disease Cardiomyopathy. *Human Immunology*.

[103] Jorge S. E. D. C., Abreu C. F., Guariento M. E., de Fatima Sonati M. (2010). Haptoglobin Genotypes in Chagas’ Disease. *Clinical Biochemistry*.

[104] Fernández N. M., Fernández-Mestre M. (2014). The Role of Haptoglobin Genotypes in Chagas Disease. *Disease Markers*.

[105] Casares-Marfil D., Strauss M., Bosch-Nicolau P. (2021). A Genome-Wide Association Study Identifies Novel Susceptibility *loci* in Chronic Chagas Cardiomyopathy. *Clinical Infectious Diseases*.

[106] Clipman S. J., Henderson-Frost J., Fu K. Y. (2018). Genetic Association Study of NLRP1, CARD, and CASP1 Inflammasome Genes With Chronic Chagas Cardiomyopathy Among *Trypanosoma cruzi* Seropositive Patients in Bolivia. *PLoS ONE*.

[107] Sandri T. L., Andrade F. A., Lidani K. C. F. (2019). Human Collectin-11 (COLEC11) and Its Synergic Genetic Interaction With MASP2 Are Associated With the Pathophysiology of Chagas Disease. *PLOS Neglected Tropical Diseases*.

[108] Sandri T. L., Lidani K. C. F., Andrade F. A. (2018). Human Complement Receptor Type 1 (CR1) Protein Levels and Genetic Variants in Chronic Chagas Disease. *Scientific Reports*.

[109] Dias F. C., da S. Medina T., Mendes-Junior C. T. (2013). Polymorphic Sites at the Immunoregulatory CTLA-4 Gene Are Associated With Chronic Chagas Disease and Its Clinical Manifestations. *PLoS ONE*.

[110] Fernández-Mestre M., Sánchez K., Balbás O. (2009). Influence of CTLA-4 Gene Polymorphism in Autoimmune and Infectious Diseases. *Human Immunology*.

[111] Luz P. R., Boldt A. B. W., Grisbach C. (2013). Association of L-Ficolin Levels and FCN2 Genotypes With Chronic Chagas Disease. *PLoS ONE*.

[112] Cavalcanti E. O., Lidani K. C. F., de Freitas Oliveira Toré C., de Messias Reason I. J., Andrade F. A. (2022). *MASP1* Gene Polymorphism and MASP-3 Serum Levels in Patients With Chronic Chagas Disease. *Immunological Investigations*.

[113] Rodriguez D. A. L., González C. I., Martin J. (2016). Analysis of Association of *FOXO3* Gene With *Trypanosoma cruzi* Infection and Chronic Chagasic Cardiomyopathy. *HLA*.

[114] da Silva Cruz G., Angelo A. L. D., Larocca T. F. (2015). Assessment of Galectin-3 Polymorphism in Subjects With Chronic Chagas Disease. *Arquivos Brasileiros de Cardiologia*.

[115] Ayo C. M., Bestetti R. B., de Campos Junior E. (2021). MICA and KIR: Immunogenetic Factors Influencing Left Ventricular Systolic Dysfunction and Digestive Clinical Form of Chronic Chagas Disease. *Frontiers in Immunology*.

[116] Weitzel T., Zulantay I., Danquah I. (2012). Mannose-Binding Lectin and Toll-Like Receptor Polymorphisms and Chagas Disease in Chile. *The American Society of Tropical Medicine and Hygiene*.

[117] Boldt A. B. W., Luz P. R., Messias-Reason I. J. T. (2011). MASP2 Haplotypes Are Associated With High Risk of Cardiomyopathy in Chronic Chagas Disease. *Clinical Immunology*.

[118] Ramasawmy R., Cunha-Neto E., Fae K. C. (2009). Heterozygosity for the S180L Variant of MAL/TIRAP, A Gene Expressing an Adaptor Protein in the Toll-Like Receptor Pathway, Is Associated With Lower Risk of Developing Chronic Chagas Cardiomyopathy. *The Journal of Infectious Diseases*.

[119] Ayo C. M., Reis P. G., de Oliveira Dalalio M. M. (2015). Killer Cell Immunoglobulin-Like Receptors and Their HLA Ligands Are Related With the Immunopathology of Chagas Disease. *PLoS Neglected Tropical Diseases*.

[120] Ayo C. M., de Oliveira A. P., da Silveira Camargo A. V., de Mattos C. C. B., Bestetti R. B., de Mattos L. C. (2015). Association of the Functional MICA-129 Polymorphism With the Severity of Chronic Chagas Heart Disease. *Clinical Infectious Diseases*.

[121] Calzada J. E., Nieto A., López-Nevot M. A., Martín J. (2001). Lack of Association Between NRAMP1 Gene Polymorphisms and *Trypanosoma cruzi* infection. *Tissue Antigens*.

[122] Ramasawmy R., Faé K. C., Cunha-Neto E. (2008). Variants in the Promoter Region of IKBL/NFKBIL1 Gene May Mark Susceptibility to the Development of Chronic Chagas’ Cardiomyopathy among Trypanosoma Cruzi-Infected Individuals. *Molecular Immunology*.

[123] Robledo G., González C. I., Morillo C., Martín J., González A. (2007). Association Study of *PTPN22* C1858T Polymorphism in *Trypanosoma cruzi* Infection. *Tissue Antigens*.

[124] Zafra G., Flórez O., Morillo C. A., Echeverría L. E., Martín J., González C. I. (2008). Polymorphisms of Toll-Like Receptor 2 and 4 Genes in Chagas Disease. *Memórias do Instituto Oswaldo Cruz*.

[125] Sánchez G., Salazar-Alcalá E., Hernández F. (2022). Polymorphisms of the TLR4 Gene: Risk Factor for Chronicity and Severity in Oral Vectorial Chagas Disease. *Experimental Parasitology*.

[126] Rodriguez D. A. L., Acosta-Herrera M., Carmona F. D. (2018). Comprehensive Analysis of Three TYK2 Gene Variants in the Susceptibility to Chagas Disease Infection and Cardiomyopathy. *PLoS ONE*.

[127] Silva M. C., Fuzo C. A., Paiva I. M. (2022). Synonymous Mutation rs1129293 Is Associated With PIK3CG Expression and PI3K*γ* Activation in Patients With Chronic Chagas Cardiomyopathy. *Immunobiology*.

[128] Fu K. Y.-J., Zamudio R., Henderson-Frost J. (2017). Association of Caspase-1 Polymorphisms With Chagas Cardiomyopathy Among Individuals in Santa Cruz, Bolivia. *Revista da Sociedade Brasileira de Medicina Tropical*.

[129] Messias-Reason I. J., Urbanetz L., Pereira da Cunha C. (2003). Complement C3 F and BF S Allotypes Are Risk Factors for Chagas Disease Cardiomyopathy. *Tissue Antigens*.

[130] Ramasawmy R., Cunha-Neto E., Faé K. C. (2006). *BAT1*, A Putative Anti-Inflammatory Gene, Is Associated With Chronic Chagas Cardiomyopathy. *The Journal of Infectious Diseases*.

[131] Galvão Da Silva A. P., Jacysyn J. F., De Almeida Abrahamsohn I. (2003). Resistant Mice Lacking Interleukin-12 Become Susceptible to *Trypanosoma cruzi* Infection but Fail to Mount a T Helper Type 2 Response. *Immunology*.

[132] Michailowsky V., Silva N. M., Rocha C. D., Vieira L. Q., Lannes-Vieira J., Gazzinelli R. T. (2001). Pivotal Role of Interleukin-12 and Interferon-*γ* Axis in Controlling Tissue Parasitism and Inflammation in the Heart and Central Nervous System During *Trypanosoma cruzi* Infection. *The American Journal of Pathology*.

[133] Hunter C. A., Ellis-Neyes L. A., Slifer T. (1997). IL-10 Is Required to Prevent Immune Hyperactivity During Infection With Trypanosoma cruzi. *The Journal of Immunology*.

[134] Oliveira A. C., Gomes-Neto J. F., Barbosa C. D. (2017). Crucial Role for T Cell-Intrinsic IL-18R-MyD88 Signaling in Cognate Immune Response to Intracellular Parasite Infection. *Elife*.

[135] Chandrasekar B., Mummidi S., Claycomb W. C., Mestril R., Nemer M. (2005). Interleukin-18 Is a Pro-Hypertrophic Cytokine That Acts Through a Phosphatidylinositol 3-Kinase-Phosphoinositide-Dependent Kinase-1-Akt-GATA4 Signaling Pathway in Cardiomyocytes. *Journal of Biological Chemistry*.

[136] Miyazaki Y., Hamano S., Wang S., Shimanoe Y., Iwakura Y., Yoshida H. (2010). IL-17 Is Necessary for Host Protection Against Acute-Phase *Trypanosoma cruzi* Infection. *The Journal of Immunology*.

[137] Ferreira R. R., Waghabi M. C., Bailly S. (2022). The Search for Biomarkers and Treatments in Chagas Disease: Insights From TGF-Beta Studies and Immunogenetics. *Frontiers in Cellular and Infection Microbiology*.

[138] Ferreira J. M., dos Santos B. R. C., de Moura E. L. (2023). Narrowing the Relationship Between Human CCR5 Gene Polymorphisms and Chagas Disease: Systematic Review and Meta-Analysis. *Life*.

[139] Silva M. C., Davoli-Ferreira M., Medina T. S. (2018). Canonical PI3K*γ* Signaling in Myeloid Cells Restricts *Trypanosoma cruzi* Infection and Dampens Chagasic Myocarditis. *Nature Communications*.

[140] de Carvalho R. V. H., Zamboni D. S. (2020). Inflammasome Activation in Response to Intracellular Protozoan Parasites. *Trends in Parasitology*.

[141] Delanghe J. R., Delrue C., Speeckaert R., Speeckaert M. M. (2024). Unlocking the Link Between Haptoglobin Polymorphism and Noninfectious Human Diseases: Insights and Implications. *Critical Reviews in Clinical Laboratory Sciences*.

[142] Deng X., Sabino E. C., Cunha-Neto E. (2013). Genome Wide Association Study (GWAS) of Chagas Cardiomyopathy in *Trypanosoma cruzi* Seropositive Subjects. *PLoS ONE*.

[143] Sabino E. C., Franco L. A. M., Venturini G. (2022). Genome-Wide Association Study for Chagas Cardiomyopathy Identify a New Risk Locus on Chromosome 18 Associated With an Immune-Related Protein and Transcriptional Signature. *PLOS Neglected Tropical Diseases*.

[144] Brochet P., Mouren J.-C., Hannouche L. (2023). ChagasDB: 80 Years of Publicly Available Data on the Molecular Host Response to *Trypanosoma cruzi* Infection in a Single Database. *Database*.

[145] Sanbe A., Marunouchi T., Abe T. (2013). Phenotype of Cardiomyopathy in Cardiac-Specific Heat Shock Protein B8 K141N Transgenic Mouse. *Journal of Biological Chemistry*.

[146] Ouarhache M., Marquet S., Frade A. F. (2021). Rare Pathogenic Variants in Mitochondrial and Inflammation-Associated Genes May Lead to Inflammatory Cardiomyopathy in Chagas Disease. *Journal of Clinical Immunology*.

[147] Kohda M., Tokuzawa Y., Kishita Y. (2016). A Comprehensive Genomic Analysis Reveals the Genetic Landscape of Mitochondrial Respiratory Chain Complex Deficiencies. *PLoS Genetics*.

[148] Scaglia F., Towbin J. A., Craigen W. J. (2004). Clinical Spectrum, Morbidity, and Mortality in 113 Pediatric Patients With Mitochondrial Disease. *Pediatrics*.

[149] Finsterer J., Frank M. (2017). Gastrointestinal Manifestations of Mitochondrial Disorders: A Systematic Review. *Therapeutic Advances in Gastroenterology*.

[150] Fang J. X., Uchiumi T., Yagi M. (2013). Dihydro-Orotate Dehydrogenase Is Physically Associated With the Respiratory Complex and Its Loss Leads to Mitochondrial Dysfunction. *Bioscience Reports*.

[151] Lee H. J., Oh Y. K., Rhee M. (2007). The Role of STAT1/IRF-1 on Synergistic ROS Production and Loss of Mitochondrial Transmembrane Potential During Hepatic Cell Death Induced by LPS/d-GalN. *Journal of Molecular Biology*.

[152] Mariappan N., Elks C. M., Fink B., Francis J. (2009). TNF-Induced Mitochondrial Damage: A Link Between Mitochondrial Complex I Activity and Left Ventricular Dysfunction. *Free Radical Biology and Medicine*.

[153] Ashour D., Rebs S., Arampatzi P. (2023). An Interferon Gamma Response Signature Links Myocardial Aging and Immunosenescence. *Cardiovascular Research*.

[154] Gallardo F., Brochet P., Goudenège D. (2023). Mitochondrial DNA Haplogroups and Variants Predispose to Chagas Disease Cardiomyopathy. *Hearts*.

[155] Martínez-Redondo D., Marcuello A., Casajús J. A. (2010). Human Mitochondrial Haplogroup H: The Highest VO2Max Consumer—Is It a Paradox?. *Mitochondrion*.

[156] McManus M. J., Picard M., Chen H.-W. (2019). Mitochondrial DNA Variation Dictates Expressivity and Progression of Nuclear DNA Mutations Causing Cardiomyopathy. *Cell Metabolism*.

[157] Atici A. E., Crother T. R., Noval Rivas M. (2023). Mitochondrial Quality Control in Health and Cardiovascular Diseases. *Frontiers in Cell and Developmental Biology*.

[158] Wan X., Garg N. J. (2021). Sirtuin Control of Mitochondrial Dysfunction, Oxidative Stress, and Inflammation in Chagas Disease Models. *Frontiers in Cellular and Infection Microbiology*.

[159] Nunes J. P. S., Roda V. M. de P., Andrieux P., Kalil J., Chevillard C., Cunha-Neto E. (2023). Inflammation and Mitochondria in the Pathogenesis of Chronic Chagas Disease Cardiomyopathy. *Experimental Biology and Medicine*.

[160] Vyatkina G., Bhatia V., Gerstner A., Papaconstantinou J., Garg N. (2004). Impaired Mitochondrial Respiratory Chain and Bioenergetics During Chagasic Cardiomyopathy Development. *Biochimica et Biophysica Acta (BBA) - Molecular Basis of Disease*.

[161] Nunes J. P. S., Andrieux P., Brochet P. (2021). Co-Exposure of Cardiomyocytes to IFN-*γ* and TNF-*α* Induces Mitochondrial Dysfunction and Nitro-Oxidative Stress: Implications for the Pathogenesis of Chronic Chagas Disease Cardiomyopathy. *Frontiers in Immunology*.

[162] Wen J. J., Porter C., Garg N. J. (2017). Inhibition of NFE2L2-Antioxidant Response Element Pathway by Mitochondrial Reactive Oxygen Species Contributes to Development of Cardiomyopathy and Left Ventricular Dysfunction in Chagas Disease. *Antioxidants & Redox Signaling*.

[163] Wen J.-J., Vyatkina G., Garg N. (2004). Oxidative Damage During Chagasic Cardiomyopathy Development: Role of Mitochondrial Oxidant Release and Inefficient Antioxidant Defense. *Free Radical Biology and Medicine*.

[164] Wen J. J., Garg N. J., Tanowitz H. B. (2018). Manganese Superoxide Dismutase Deficiency Exacerbates the Mitochondrial ROS Production and Oxidative Damage in Chagas Disease. *PLOS Neglected Tropical Diseases*.

[165] Teixeira P. C., Ducret A., Langen H. (2021). Impairment of Multiple Mitochondrial Energy Metabolism Pathways in the Heart of Chagas Disease Cardiomyopathy Patients. *Frontiers in Immunology*.

[166] Correia M., Santos F., da Silva Ferreira R., Ferreira R., Bernardes de Jesus B., Nóbrega-Pereira S. (2022). Metabolic Determinants in Cardiomyocyte Function and Heart Regenerative Strategies. *Metabolites*.

[167] Piquereau J., Caffin F., Novotova M. (2013). Mitochondrial Dynamics in the Adult Cardiomyocytes: Which Roles for a Highly Specialized Cell?. *Frontiers in Physiology*.

[168] Lopaschuk G. D., Karwi Q. G., Tian R., Wende A. R., Abel E. D. (2021). Cardiac Energy Metabolism in Heart Failure. *Circulation Research*.

[169] Pascual F., Coleman R. A. (2016). Fuel Availability and Fate in Cardiac Metabolism: A Tale of Two Substrates. *Biochimica et Biophysica Acta (BBA) - Molecular and Cell Biology of Lipids*.

[170] Piquereau J., Ventura-Clapier R. (2018). Maturation of Cardiac Energy Metabolism During Perinatal Development. *Frontiers in Physiology*.

[171] Ng S. M., Neubauer S., Rider O. J. (2023). Myocardial Metabolism in Heart Failure. *Current Heart Failure Reports*.

[172] Lim G. B. (2020). Inhibiting Fatty Acid Oxidation Promotes Cardiomyocyte Proliferation. *Nature Reviews Cardiology*.

[173] Kiyuna L. A., Candido D. S., Bechara L. R. G. (2023). 4-Hydroxynonenal Impairs miRNA Maturation in Heart Failure via Dicer Post-Translational Modification. *European Heart Journal*.

[174] Leme A. M. B. P., Salemi V. M. C., Parga J. R. (2010). Evaluation of the Metabolism of High Energy Phosphates in Patients With Chagas’ Disease. *Arquivos Brasileiros de Cardiologia*.

[175] Teixeira P. C., Santos R. H. B., Fiorelli A. I. (2011). Selective Decrease of Components of the Creatine Kinase System and ATP Synthase Complex in Chronic Chagas Disease Cardiomyopathy. *PLoS Neglected Tropical Diseases*.

[176] Díaz M. L., Burgess K., Burchmore R. (2022). Metabolomic Profiling of End-Stage Heart Failure Secondary to Chronic Chagas Cardiomyopathy. *International Journal of Molecular Sciences*.

[177] Palomino S. A. P., Aiello V. D., Higuchi M. L. (2000). Systematic Mapping of Hearts From Chronic Chagasic Patients: the Association Between the Occurrence of Histopathological Lesions and *Trypanosoma cruzi* Antigens. *Annals of Tropical Medicine & Parasitology*.

[178] Zorova L. D., Popkov V. A., Plotnikov E. Y. (2018). Mitochondrial Membrane Potential. *Analytical Biochemistry*.

[179] Lkhagva B., Kao Y.-H., Lee T.-I., Lee T.-W., Cheng W.-L., Chen Y.-J. (2018). Activation of Class I Histone Deacetylases Contributes to Mitochondrial Dysfunction in Cardiomyocytes With Altered Complex Activities. *Epigenetics*.

[180] Lee T.-I., Kao Y.-H., Baigalmaa L. (2019). Sodium Hydrosulphide Restores Tumour Necrosis Factor-*α*-Induced Mitochondrial Dysfunction and Metabolic Dysregulation in HL-1 Cells. *Journal of Cellular and Molecular Medicine*.

[181] Wan X., Wen J.-J., Koo S.-J., Liang L. Y., Garg N. J., Bozza M. T. (2016). SIRT1-PGC1*α*-NF*κ*B Pathway of Oxidative and Inflammatory Stress During *Trypanosoma cruzi* Infection: Benefits of SIRT1-Targeted Therapy in Improving Heart Function in Chagas Disease. *PLoS Pathogens*.

[182] Van Linthout S., Tschöpe C. (2017). Inflammation—Cause or Consequence of Heart Failure or Both?. *Current Heart Failure Reports*.

[183] Bosch-Nicolau P., Fernández M. L., Sulleiro E. (2024). Efficacy of Three Benznidazole Dosing Strategies for Adults Living With Chronic Chagas Disease (MULTIBENZ): An International, Randomised, Double-Blind, Phase 2b Trial. *The Lancet Infectious Diseases*.

[184] Messenger A. G., Rundegren J. (2004). Minoxidil: Mechanisms of Action on Hair Growth. *British Journal of Dermatology*.

[185] Storz U. (2014). Rituximab: How Approval History Is Reflected by a Corresponding Patent Filing Strategy. *mAbs*.

[186] Michelson D., Faries D., Wernicke J. (2001). Atomoxetine in the Treatment of Children and Adolescents With Attention-Deficit/Hyperactivity Disorder: A Randomized, Placebo-Controlled, Dose-Response Study. *Pediatrics*.

[187] Wolraich M. L., Hagan J. F., Allan C. (2019). Clinical Practice Guideline for the Diagnosis, Evaluation, and Treatment of Attention-Deficit/Hyperactivity Disorder in Children and Adolescents. *Pediatrics*.

[188] Wu Q., Wang Q., Mao G., Dowling C. A., Lundy S. K., Mao-Draayer Y. (2017). Dimethyl Fumarate Selectively Reduces Memory T Cells and Shifts the Balance Between Th1/Th17 and Th2 in Multiple Sclerosis Patients. *The Journal of Immunology*.

[189] Jourdan J.-P., Bureau R., Rochais C., Dallemagne P. (2020). Drug Repositioning: A Brief Overview. *Journal of Pharmacy and Pharmacology*.

[190] Roessler H. I., Knoers N. V. A. M., van Haelst M. M., van Haaften G. (2021). Drug Repurposing for Rare Diseases. *Trends in Pharmacological Sciences*.

[191] Pandey R. P., Nascimento M. S., Moore C. E., Raj V. S., Kalil J., Cunha-Neto E. (2021). New Approaches for the Treatment of Chagas Disease. *Current Drug Targets*.

[192] Pandey R. P., Nascimento M. S., Franco C. H. (2022). Drug Repurposing in Chagas Disease: Chloroquine Potentiates Benznidazole Activity Against *Trypanosoma cruzi In Vitro* and *In Vivo*. *Antimicrobial Chemotherapy*.

[193] Molina I., Gómez i Prat J., Salvador F. (2014). Randomized Trial of Posaconazole and Benznidazole for Chronic Chagas’ Disease. *New England Journal of Medicine*.

[194] Lannes-Vieira J. (2022). Multi-Therapeutic Strategy Targeting Parasite and Inflammation-Related Alterations to Improve Prognosis of Chronic Chagas Cardiomyopathy: A Hypothesis-Based Approach. *Memórias do Instituto Oswaldo Cruz*.

[195] Bilate A. M. B., Salemi V. M., Ramires F. J. (2007). TNF Blockade Aggravates Experimental Chronic Chagas Disease Cardiomyopathy. *Microbes and Infection*.

[196] Rolski F., Tkacz K., Węglarczyk K. (2024). TNF-*α* Protects From Exacerbated Myocarditis and Cardiac Death by Suppressing Expansion of Activated Heart-Reactive CD4+ T Cells. *Cardiovascular Research*.

[197] Wen J.-J., Gupta S., Guan Z. (2010). Phenyl-*α*-tert-butyl-nitrone and Benzonidazole Treatment Controlled the Mitochondrial Oxidative Stress and Evolution of Cardiomyopathy in Chronic Chagasic Rats. *Journal of the American College of Cardiology*.

[198] Vilar-Pereira G., Carneiro V. C., Mata-Santos H. (2016). Resveratrol Reverses Functional Chagas Heart Disease in Mice. *PLOS Pathogens*.

[199] Cevey Á.C., Mirkin G. A., Donato M. (2017). Treatment With Fenofibrate Plus a Low Dose of Benznidazole Attenuates Cardiac Dysfunction in Experimental Chagas Disease. *International Journal for Parasitology: Drugs and Drug Resistance*.

[200] Tanaka D. M., Fabricio C. G., Marin-Neto J. A. (2023). Pentoxifylline Reduces Inflammation and Prevents Myocardial Perfusion Derangements in Experimental Chronic Chagas’ Cardiomyopathy. *Journal of Nuclear Cardiology*.

[201] Silva Grijó Farani P., Iandra da Silva Ferreira B., Begum K. (2023). Treatment With Benznidazole and Pentoxifylline Regulates microRNA Transcriptomic Profile in a Murine Model of Chagas Chronic Cardiomyopathy. *PLOS Neglected Tropical Diseases*.

[202] Reinfuss M. (1987). [Kaposi’s Sarcoma. Clinical Picture, Treatment and Prognosis. I. Clinical Forms of Kaposi’s Sarcoma]. *Nowotwory*.

[203] Ramires F. J. A., Salemi V. M. C., Ianni B. M. (2006). Aldosterone Antagonism in an Inflammatory State: Evidence for Myocardial Protection. *Journal of the Renin-Angiotensin-Aldosterone System*.

[204] Fernandes F., Ramires F. J. A., Ianni B. M. (2012). Effect of Colchicine on Myocardial Injury Induced by *Trypanosoma cruzi* in Experimental Chagas Disease. *Journal of Cardiac Failure*.

[205] Krcek L., Novák M., Vávrová M., Hovorková A. (1989). [Uptake of Osteotropic Agents in the Spine in Laboratory Rats After the Administration of Biphenyl Derivatives]. *Bratislavske lekarske listy*.

